# Exploring Phenolic Compounds Extraction from Saffron (*C. sativus*) Floral By-Products Using Ultrasound-Assisted Extraction, Deep Eutectic Solvent Extraction, and Subcritical Water Extraction

**DOI:** 10.3390/molecules29112600

**Published:** 2024-06-01

**Authors:** Valentina Masala, Stela Jokić, Krunoslav Aladić, Maja Molnar, Carlo Ignazio Giovanni Tuberoso

**Affiliations:** 1 Department of Life and Environmental Sciences, University of Cagliari, Cittadella Universitaria di Monserrato, S.P. Monserrato-Sestu km 0.700, 09042 Monserrato, Italy; valentina.masala2@unica.it; 2 Faculty of Food Technology Osijek, Josip Juraj Strossmayer University of Osijek, Franje Kuhača 18, 31000 Osijek, Croatia; kaladic@ptfos.hr (K.A.); mmolnar@ptfos.hr (M.M.)

**Keywords:** saffron, floral by-product, green extraction, HPLC-PDA, LC-MS/MS, phenolic compounds, delphinidin 3,5-di-*O*-glucoside, kaempferol 3-*O*-sophoroside

## Abstract

Saffron (*Crocus sativus*) floral by-products are a source of phenolic compounds that can be recovered and used in the nutraceutical, pharmaceutical, or cosmetic industries. This study aimed to evaluate the phenolic compounds’ extraction using green extraction techniques (GETs) in saffron floral by-products and to explore the influence of selected extraction techniques on the phytochemical composition of the extracts. Specifically, ultrasound-assisted extraction (UAE), subcritical water extraction (SWE), and deep eutectic solvents extraction (DESE) were used. Phenolic compounds were identified with (HR) LC-ESI-QTOF MS/MS analysis, and the quantitative analysis was performed with HPLC-PDA. Concerning the extraction techniques, UAE showed the highest amount for both anthocyanins and flavonoids with 50:50% *v*/*v* ethanol/water as solvent (93.43 ± 4.67 mg/g of dry plant, dp). Among SWE, extraction with 96% ethanol and t = 125 °C gave the best quantitative results. The 16 different solvent mixtures used for the DESE showed the highest amount of flavonoids (110.95 ± 5.55–73.25 ± 3.66 mg/g dp), while anthocyanins were better extracted with choline chloride:butane-1,4-diol (16.0 ± 0.80 mg/g dp). Consequently, GETs can be employed to extract the bioactive compounds from saffron floral by-products, implementing recycling and reduction of waste and fitting into the broader circular economy discussion.

## 1. Introduction

Saffron (*Crocus sativus* L.) is a traditional perennial plant commonly used as a spice, a natural colorant in food, and a flavoring agent. The *C. sativus* flower is composed of three golden yellow stamens, six purple tepals, and one red pistil. The pistil ends with three red, branched stigmas, whose length surpasses that of the tepals and which, when dried up, represent the saffron spice. Although saffron is widely produced in Italy, Greece, Spain, and other Mediterranean countries, Iran is the primary supplier, with 111,000 hectares of saffron farms and about 404 tons of production in 2018 [1]. Only the flower stigmas are used for saffron production, while the tepals and stamens are simply discarded. It is estimated that for the production of 1 kg of saffron spice, around 350 kg of saffron floral by-products (SFBPs) are produced [2].

The current production system, such as harvesting and processing, generates huge amounts of waste with a high environmental impact. This saffron waste can be used as a source of bioactive compounds and, moreover, finds its application in the health and food industries. Food by-products have untapped potential in the context of functional foods. By valorizing these by-products, we conform to circular economy principles, aiming to protect the environment and promote economic development [3].

Both the saffron stigmas and the floral bio-residues have a high fiber content, are rich in carbohydrates and proteins, and contain a low amount of fat; they also have high concentrations of glucose; fructose; lactic and malic acids; and minerals like potassium, calcium, and magnesium [4]. It is notable that several phenolic compounds have been detected in SFBPs, especially anthocyanins and flavonoids. Regarding anthocyanins, delphinidin 3,5-di-*O*-glucoside is the most abundant one, followed by delphinidin 3-*O*-glucoside, petunidin 3,5-di-*O*-glucoside, and petunidin 3-*O*-glucoside [5,6]. It has been demonstrated that delphinidin 3,5-di-*O*-glucoside functions to control the lacrimal gland’s production of tears [6]. Among flavonoids, the most abundant is kaempferol 3-*O*-sophoroside [5,6], which is known for several biological activities such as anti-inflammatory, antiradical, and antioxidant activity [6,7,8,9]. The ABTS^•+^ technique was used to demonstrate the substantial antioxidant activity of these bio-residue components and to highlight the variations in extracts derived from various saffron plant parts [10]. 

Conventional extraction techniques for SFBPs have been performed, including Soxhlet extraction [11], maceration with different solvents like acidic ethanol [12], ethanol at different solvent ratios [13,14], ethanol/water [15], methanol [16], and microwave-assisted extraction (MAE) [17,18]. Concerning the environmental issues related to this huge waste production, in recent times green extraction techniques (GETs) have spread as an eco-friendly and sustainable way to obtain plant extracts that contain bioactive compounds of interest. Generally recognized as safe (GRAS) and safer choice standard solvents ensure that GETs employ environmentally friendly solvents and optimize extraction cycles by minimizing energy consumption and sample deterioration [19]. Different GETs were used to recover these molecules because *C. sativus* produces a lot of floral by-products and because these by-products contain valuable bioactive components. Among GETs, ultrasound-assisted extraction (UAE) and subcritical water extraction (SWE) were tested for SFBPs in previous studies. UAE in an ultrasonic bath, which is a conventional method, has been frequently used. It was performed with different solvent mixtures and ratios, such as EtOH:H_2_O [20,21], and with deionized H_2_O or MeOH in deionized water at different ratios [22]. A more innovative UAE extraction technique was performed with an ultrasound sonotrode with distilled water and NaCl [23] and EtOH:H_2_O as solvent mixtures at different ratios [24]. Based on our information, UAE with an ultrasound sonotrode, monitoring amplitude and impulse and comparing different solvents (100% H_2_O, 50:50% *v*/*v* EtOH:H_2_O, and 96:4% *v*/*v* EtOH:H_2_O), has not been performed yet on SFBPs. Instead, SWE refers to the use of H_2_O in its subcritical state (obtained with both high temperature and high pressure), causing a decrease in its polarity and making it behave similarly to MeOH or EtOH [25]. Compared with organic solvents, subcritical water not only has advantages in ecology, economy, and safety, but also in terms of density, ion product, and dielectric constant that can be adjusted by temperature [25]. Those distinctive characteristics make subcritical water suitable for the extraction of herbs, vegetables, and fruits [26,27]. SWE is a type of pressurized liquid extraction (PLE) using water as the solvent. PLE with different green solvents (distilled water, citric acid, and lactic acid at different ratios) was tested on SFBPs, but it provided inferior results compared with other techniques (stirred-tank extraction, stirred-tank extraction with ultrasonication pretreatment), producing extracts with lower polyphenolic concentrations and weaker antioxidant properties [28]. To the best of our knowledge, the comparison of SWE/PLE with 100% H_2_O, EtOH:H_2_O (50:50, *v*/*v),* and 96% EtOH for the extraction of phenolic compounds from SFBPs has not been described before.

Finally, regarding the search for new green solvents to replace the traditional organic ones, the use of deep eutectic solvents (DESs) has recently gained popularity in polyphenol extraction. DESs are a mixture of a hydrogen bond acceptor (HBA) and a hydrogen bond donor (HBD); they show many advantages, such as biodegradability, low toxicity, and ease of handling [29]. The most common DESs are formed by choline chloride (ChCl) with a cheap and safe HBD such as urea, ethylene glycol, and glycerol, but other alcohols, amino acids, carboxylic acids, and sugars have also been commonly used [29]. As far as we know, DESE (DES extraction) has never been performed in SFBPs, but natural DESs (NaDESs), obtained by combining molecules copiously present in nature, have been used. Indeed, it is important to highlight that NaDESs, which are mostly polar, can co-extract some elements from plant materials. Recent research demonstrated that there was no health or carcinogenic risk following topical application of the tested NaDESs, with the calculated daily intake of trace elements from the NaDES extracts being below the daily dose risk estimators. This suggests that NaDESs can have yet another important advantage over other solvents [30]. For this purpose, Lakka et al. [31] performed an NaDES extraction technique assisted by a batch-stirred tank extractor, using as a naturally derived solvent a eutectic mixture of L-lactic acid (HBD) and glycine (HBA) (5:1 ratio), which proved to be green and efficient. It produced extracts rich in flavonols and anthocyanins with strong antioxidant properties. Moreover, in the study of Cerda-Bernad et al. [32], in which the potential of chitosan and alginate base hydrogels as carriers for phenolic compounds was explored, the authors used NaDESs combined with UAE. Furthermore, NaDESs have been proposed as a method to increase the bioavailability of other natural bioactive compounds in addition to polyphenols, such as quassinoids and alkaloids from *Eurycoma longifolia* [33], triterpene saponins from *Aralia elata* [34], polysaccharides from *Auricularia auricula* [35], and phlorotannins from *Fucus vesiculosus* [36]. 

Taking into account the previous experiments performed so far, this study investigated the variation in the phenolic compounds of extracts from floral by-products of *C. sativus* L. obtained with different GETs. For this purpose, UAE with ultrasound sonotrodes at different values of amplitude and impulse and H_2_O:EtOH ratio, DESE with ChCl as HBA and different organic compounds as HBDs, and SWE at different temperatures and H_2_O:EtOH ratios, were performed. Moreover, UAE extracts were further investigated using response surface methodology (RSM). (HR) LC-ESI-QTOF MS/MS in negative and positive ion modes and HPLC-PDA analysis were used for the quali-quantitative investigation of phenolic compounds in the GETs extracts from the SFBPs.

## 2. Results and Discussion

*C. sativus* floral by-products were extracted with three different GETs set with different parameters (Table 1), and the polyphenolic composition of the obtained extracts was investigated using LC-MS/MS and LC-PDA. A total of 16 samples were used for UAE, 14 for SWE, and 16 for DESE, based on the authors’ previous experience [37,38,39].

### 2.1. Qualitative Determination of Phenolic Compounds in C. sativus Extracts

**Figure 1:**
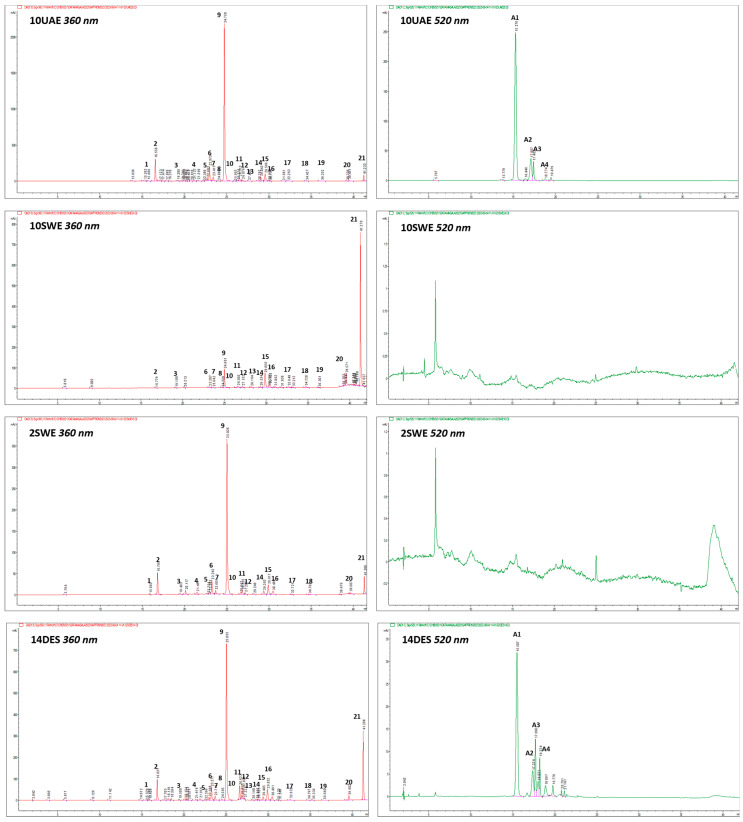
HPLC-PDA fingerprinting for selected *C. sativus* floral by-product extracts (UAE: ultrasound-assisted extraction; SWE: subcritical water extraction; DES: deep eutectic solvent) at λ = 360 and 520 nm. Peak identification is given in Table 2. Chromatographic conditions are described in the text.

The *C. sativus* floral by-product extracts were qualitatively analyzed using (HR) LC-ESI-QTOF MS/MS in negative and positive ion modes, and the targeted phenolic compounds were quantified by HPLC-PDA analysis (Figure 1).

Table 2 reports the phenolic compounds detected in the floral by-product extracts, listed according to their LC-PDA retention times. Compounds were identified using (HR) LC-ESI-QTOF MS/MS in negative and positive ion modes, and the molecular formula derived by mass measurement (experimental result); MS/MS results, mass error (Δ ppm), the references used for identification, and the identification confidence levels [40] are reported. Twenty-five compounds were identified by comparing the *m*/*z* values with those described in the literature and by comparing experimental MS/MS spectra with the fragmentation patterns reported in the literature or with the fragmentation patterns and spectra reported in a public repository of mass spectral data [41,42]. Compounds **1**–**21** were attributed to flavonoids (mainly kaempferol, myricetin, isorhamnetin, and quercetin derivatives), and Compounds **A1**–**A4** to anthocyanins (mainly delphinidin and petunidin derivatives).

Compound **1** was identified as kaempferol sophoroside-glucoside due to the [M-H]^−^ at *m*/*z* 771.1999 with fragments at *m*/*z* 609.1441 (loss of a kaempferol diglucoside unit), 285.0382 (loss of a kaempferol unit), 284.0300, and 283.0243 and the [M+H]^+^ at *m*/*z* 773.2142 with fragments at *m*/*z* 287.0553. Compound **2** was identified as kaempferol tri-*O*-glucoside due to the [M-H]^−^ at *m*/*z* 771.2004 with fragments at *m*/*z* 609.1447 (loss of a kaempferol diglucoside unit), 284.0847, 283.0236, and 285.0354 (loss of a kaempferol unit) and due to the [M+H^]+^ at *m*/*z* 773.2014 and a fragment at *m*/*z* 287.0546; both Compounds **1** and **2** were identified through a comparison with previous studies [6,43,44]. Compound **3** was tentatively identified as kaempferol tri-*O*-glucoside with an acetyl moiety due to the [M-H]^−^ at *m*/*z* 813.2088 with fragments at *m*/*z* 651.1588, 285.0396 (loss of a kaempferol unit), 284.0322, and 283.0240; the [M+H]^+^ at *m*/*z* 815.2234 with fragments at 287.0542; and comparison with the literature data [43]. Peak **4** was attributed to myricetin glucosyl-glucoside due to the [M-H]^−^ at *m*/*z* 641.1354 with a fragment at *m*/*z* 316.0215, the [M+H]^+^ at *m*/*z* 643.1504 and a fragment at *m*/*z* 319.0450, and comparison with the literature data [43]. Compounds **5** and **6** showed similar [M-H]^−^ at, respectively, *m*/*z* 625.1415 with fragments at *m*/*z* 301.0335 (loss of a quercetin unit), 300.0271, and 271.0299 and *m*/*z* 625.1409 with fragments at 463.0375, 300.0261, and 301.0346 and the [M+H]^+^ at, respectively, *m*/*z* 627.1565 with fragments at *m*/*z* 303.0499 and *m*/*z* 627.1568 with fragments at 303.0500. They were tentatively attributed, respectively, to quercetin diglucoside and quercetin sophoroside by comparison with the literature data [6,43,44]. Peak **7** was tentatively attributed to kaempferol di-*O*-glucoside, with a molecular formula C_27_H_30_O_16_, due to the [M-H]^−^ at *m*/*z* 609.1465 with fragments at *m*/*z* 285.0391 (loss of a kaempferol unit) and 284.0323, the [M+H]^+^ at *m*/*z* 611.1606 with a fragment at 287.0550, and comparison with the literature data [43]. Compound **8** was identified as isorhamnetin di-*O*-glucoside due to the [M-H]^−^ at *m*/*z* 639.1568 with fragments at *m*/*z* 313.0339, the [M+H]^+^ at *m*/*z* 641.1716 with a fragment at *m*/*z* 317.0658, and comparison with previous studies [6,43,44]. The tallest peak (**9**) was identified as kaempferol 3-*O*-sophoroside due to the [M-H]^−^ at *m*/*z* 609.1463 with fragments at m/z 285.0386 (loss of a kaempferol unit), 284.0325, and 255.0291 and the [M+H]^+^ at *m*/*z* 611.1615 with a fragment at *m*/*z* 287.0555. Furthermore, it was confirmed by the injection of the standard and comparison with the literature data [6,43,44]. Compound **10** was tentatively identified as isorhamnetin sophoroside with the molecular formula C_28_H_32_O_17_. This is due to the [M-H]^−^ at *m*/*z* 639.156 with fragments at 315.0495, 314.0422, 299.0171, and 300.0261; the [M+H]^+^ at *m*/*z* 641.1710 with a fragment at *m*/*z* 317.0659; and comparison with the literature data [43]. Peak **11** was attributed to quercetin-3-*O*-glucoside due to the [M-H]^−^ at *m*/*z* 463.0878 with fragments at *m*/*z* 301.0322 (loss of a quercetin unit) and 300.0263 and the [M+H]^+^ at *m*/*z* 465.1030 with fragments at *m*/*z* 303.0501 and 85.0287. It was attributed also due to comparison with the pure standard and literature data [6,43]. Compound **12** was tentatively identified as kaempferol glucoside-rhamnose with the molecular formula C_27_H_30_O_15_ due to the [M-H]^−^ at *m*/*z* 593.1508 with a fragment at *m*/*z* 284.0312, [M+H]^+^ at *m*/*z* 595.1656 with a fragment at *m*/*z* 287.0551, and comparison with the literature data [43]. Compound **13** was tentatively identified as isorhamnetin (rhamnosyl)-glucoside with the molecular formula C_28_H_32_O_16_ due to the [M-H]^−^ at *m*/*z* 623.1618 with a fragment at *m*/*z* 314.0424, the [M+H]^+^ at *m*/*z* 625.1768 with a fragment at *m*/*z* 317.0658, and comparison with previous studies [43]. Peak **14** was attributed to kaempferol 3-*O*-glucoside due to the [M-H]^−^ at *m*/*z* 447.0935 with fragments at *m*/*z* 285.0386 (loss of a kaempferol unit), 284.0332, and 255.0305; the [M+H]^+^ at *m*/*z* 449.2088 with a fragment at 287.0543; and comparison with the pure standard and literature data [6,43,44].

**Table 2:** Compounds identified using (HR) LC-ESI-QTOF MS/MS in *C. sativus* floral by-products.

# n°	Rt min	Identity	Molecular Formula	[M-H]^−^ *m*/*z*	MS/MS * m/*z*	Δppm	[M]^+^/[M+H]^+^ *m*/*z*	MS/MS * m/*z*	Δppm	References	Level
1	15.69	Kaempferol sophoroside-glucoside	C_33_H_40_O_21_	771.1999	609.1441(80)/285.0382(80)/284.0300(100)/283.0243(100)	1.05	773.2142	287.0553(100)	0.52	[6,43,44]	2
2	16.56	Kaempferol tri-*O*-glucoside	C_33_H_40_O_21_	771.2004	609.1447(63)/284.0847(33)/283.0236(20)/285.0354(100)	−0.97	773.2014	287.0546(100)	0.55	[6,43,44]	2
3	19.40	Kaempferol acetyl tri-*O*-glucoside	C_35_H_42_O_22_	813.2088	651.1588(29)/285.0396(53)/284.0322(100)/283.0240(73)	−0.79	815.2234	287.0542(100)	−0.82	[43]	2
4	21.32	Myricetin glucosyl-glucoside	C_27_H_30_O_18_	641.1354	316.0215(100)	−0.86	643.1504	319.0450(100)	−0.2	[43]	2
5	22.69	Quercetin di-*O*-glucoside	C_27_H_30_O_17_	625.1415	301.0335(32)/300.0271(100)/271.0299(19)	0.49	627.1565	303.0499(100)	1.27	[6,43,44]	2
6	22.97	Quercetin sophoroside	C_27_H_30_O_17_	625.1409	463.0375(78)/300.0261(69)/301.0346(100)	−0.21	627.1568	303.0500(100)	1.56	[6,43,44]	2
7	23.41	Kaempferol di-*O*-glucoside	C_27_H_30_O_16_	609.1465	285.0391(32)/284.0323(100)	0.66	611.1606	287.0550(100)	−0.11	[43]	2
8	24.01	Isorhamnetin di-*O*-glucoside	C_28_H_32_O_17_	639.1568	313.0339(82)	−0.11	641.1716	317.0658(100)	0.52	[6,43,44]	2
9	24.73	Kaempferol 3-*O*-sophoroside	C_27_H_30_O_16_	609.1463	285.0386(33)/284.0325(100)/255.0291(14)	0.33	611.1615	287.0555(100)	1.37	[6,43,44]	1
10	24.80	Isorhamnetin sophoroside	C_28_H_32_O_17_	639.156	315.0495(43)/314.0422(100)/299.0171(41)/300.0261(14)	−0.81	641.1710	317.0659(100)	−0.26	[43]	2
11	25.99	Quercetin 3-*O*-glucoside	C_21_H_20_O_12_	463.0878	301.0322(33)/300.0263(100)	−1.3	465.1030	303.0501(100)/85.0287(10)	0.49	[6,43]	1
12	26.32	Kaempferol glucoside rhamnose	C_27_H_30_O_15_	593.1508	284.0312(100)	−0.8	595.1656	287.0551(100)	−0.34	[43]	2
13	26.51	Isorhamnetin (rhamnosyl)-glucoside	C_28_H_32_O_16_	623.1618	314.0424(100)	−0.16	625.1768	317.0658(100)	0.55	[43]	2
14	29.04	Kaempferol 3-*O*-glucoside	C_21_H_20_O_11_	447.0935	285.0386(53)/284.0332(100)/255.0305(33)	−0.5	449.2088	287.0543(100)	−0.12	[6,43,44]	1
15	29.57	Kaempferol acetyl di-*O*-glucoside	C_29_H_32_O_17_	651.1574	285.0385(27)/284.0322(100)/255.0285(18)	0.81	653.1725	287.0551(100)	1.63	[43]	2
16	29.99	Isorhamnetin 3-*O*-glucoside	C_22_H_22_O_12_	477.1036	314.0405(100)/315.0467(80)/271.0240(56)	−0.82	479.1191	317.0656(100)	1.32	[43]	1
17	32.26	Kaempferol acetyl glucoside	C_23_H_22_O_12_	489.1034	285.0356(38)/284.0296(100)	−0.7	491.1190	287.0549(100)	0.8	[43]	2
18	34.43	Quercetin	C_15_H_10_O_7_	301.0346	178.0970(61)/151.0012(100)/177.0557(51)	−2.88	303.0501	303.0502(100)/165.0165(13)	1.05	[6]	1
19	36.25	Quercetin coumaroyl-glucoside	C_30_H_26_O_14_	609.124	463.0971(25)/301.0343(67)/300.0261(100)	−1.65	611.1398	147.0435(100)/303.049(16)	1.00	[43]	2
20	39.45	Isorhamnetin coumaroyl-glucoside	C_31_H_28_O_14_	623.1395	315.0508(100)/314.0446(64)/258.0551(22)	−1.73	625.1547	147.0427(100)	−0.65	[43]	2
21	41.23	Kaempferol	C_15_H_10_O_6_	285.0399	285.0399 (100)	−1.98	287.0552	287.0553(100)	0.45	[6,43]	1
A1	15.05	Delphinidin 3,5-di-*O*-glucoside	C_27_H_31_O_17_	-	-	−	627.1564	465.1039	0.6	[6,43]	1
A2	17.37	Petunidin 3,5-di-*O*-glucoside	C_28_H_33_O_17_	-	-	−	641.1717	465.1044	−0.9	[6,43]	1
A3	17.77	Delphinidin 3-*O*-glucoside	C_21_H_21_O_12_	-	-	−	465.1015	303.0092	−3.2	[6,43]	1
A4	18.22	Petunidin 3-*O*-glucoside	C_22_H_23_O_12_	-	-	−	479.1108	317.2589	−1.9	[6,43]	1

* in parentheses, the relative intensity; # according to Blaženović [35].

Compound **15** was tentatively identified as kaempferol di-*O*-glucoside with an acetyl moiety with the molecular formula C_29_H_32_O_17_ due to the [M-H]^−^ at *m*/*z* 651.1574 with fragments at *m*/*z* 285.0385 (loss of a kaempferol unit), 284.0322, and 255.0285; the [M+H]^+^ at *m*/*z* 653.1725 with a fragment at *m*/*z* 287.0551; and comparison with the literature data [43]. Peak **16** was attributed to isorhamnetin-3-*O*-glucoside with the molecular formula C_22_H_22_O_12_ due to the [M-H]^−^ at *m*/*z* 477.1036 with fragments at *m*/*z* 314.0405, 315.0467 (loss of an isorhamnetin unit), and 271.0240; due to the [M+H]^+^ at *m*/*z* 479.1191 with a fragment at 317.0656; and comparison with the pure standard and literature data [43]. Peak **17** was tentatively attributed to kaempferol glucoside with an acetyl moiety with the molecular formula C_23_H_22_O_12_ due to the [M-H]^−^ at *m*/*z* 489.1034 with fragments at *m*/*z* 285.0356 (loss of a kaempferol unit) and 284.0296, due to the [M+H]^+^ at *m*/*z* 491.1190 with a fragment at 287.0549, and comparison with the literature data [43]. Compound **18** was identified as quercetin aglycone due to the [M-H]^−^ at *m*/*z* 301.0346 with fragments at 178.0970, 151.0012, and 177.0557; due to the [M+H]^+^ at *m*/*z* 303.0501 with fragments at 303.0502 and 165.0165; and due to comparison with the pure standard and literature data [6]. Peak **19** was tentatively attributed to quercetin coumaroyl-glucoside with the molecular formula C_30_H_26_O_14_ due to the [M-H]^−^ at *m*/*z* 609.124 with fragments at *m*/*z* 463.0971 (loss of a quercetin-glucoside unit), 301.0343 (loss of a quercetin unit), and 300.0261; due to the [M+H]^+^ at *m*/*z* 611.1398 with fragments at 147.0435 and 303.049; and comparison with the literature data [43]. Compound **20** was tentatively identified as isorhamnetin coumaroyl-glucoside with the molecular formula C_31_H_28_O_14_ due to the [M-H]^−^ at *m*/*z* 623.1395 with fragments at *m*/*z* 315.0508 (loss of an isorhamnetin unit), 314.0446, and 258.0551; due to the [M+H]^+^ at *m*/*z* 625.1547 with a fragment at 147.0427; and comparison with previous studies [43]. Peak **21** was attributed to kaempferol aglycone due to the [M-H]^−^ at *m*/*z* 285.0399, due to the [M+H]^+^ at *m*/*z* 287.0552, and by comparison with the pure standard and literature data [6,43].

Compounds **A1**–**A4** were identified as four anthocyanins in positive mode. Peaks **A1** and **A3** were attributed to delphinidin 3,5-di-*O*-glucoside and delphinidin 3-*O*-glucoside due to the [M+H]^+^ at *m*/*z* 627.1564 with a fragment at 465.1039 and [M+H]^+^ at *m*/*z* 465.1015 with a fragment at 303.0092, respectively, and comparison with the pure standards [6,43]. Compounds **A2** and **A4** were identified as petunidin 3,5-di-*O*-glucoside and petunidin 3-*O*-glucoside due to the [M+H]^+^ at *m*/*z* 641.1717 with a fragment at 465.1044 and [M+H]^+^ at *m*/*z* 479.1108 with a fragment at 317.2589, respectively, and comparison with the literature data and pure standards [6,43].

*C. sativus* flower by-product extracts showed substantial similarity with the literature data, confirming that the two most representative compounds are delphinidin 3,5-di-*O*-glucoside and kaempferol 3-*O*-sophoroside for anthocyanins and flavonoids, respectively [5,6,43,44]. 

Anthocyanins were detected in positive ion mode in their native forms (positive flavylium cations) [45,46], and so they are not visible in negative ion mode. However, flavonoids were detected in both positive and negative ion modes for a more complete and detailed qualitative analysis. The extracted flavonoids have several biological activities, mainly due to their strong antioxidant activity. Considering the two most prevalent substances, kaempferol 3-*O*-sophoroside has shown anti-inflammatory and anti-radical effects as well as hepatoprotective activity [9], and delphinidin 3,5-di-*O*-glucoside is involved in drug metabolism and carcinogenesis [6]. This could support the use of saffron floral by-products for health purposes.

### 2.2. Quantitative Determination of Phenolic Compounds in C. sativus Extracts and Influence of Extraction Technique on Selected Phenolic Compounds’ Content

**Figure 2:**
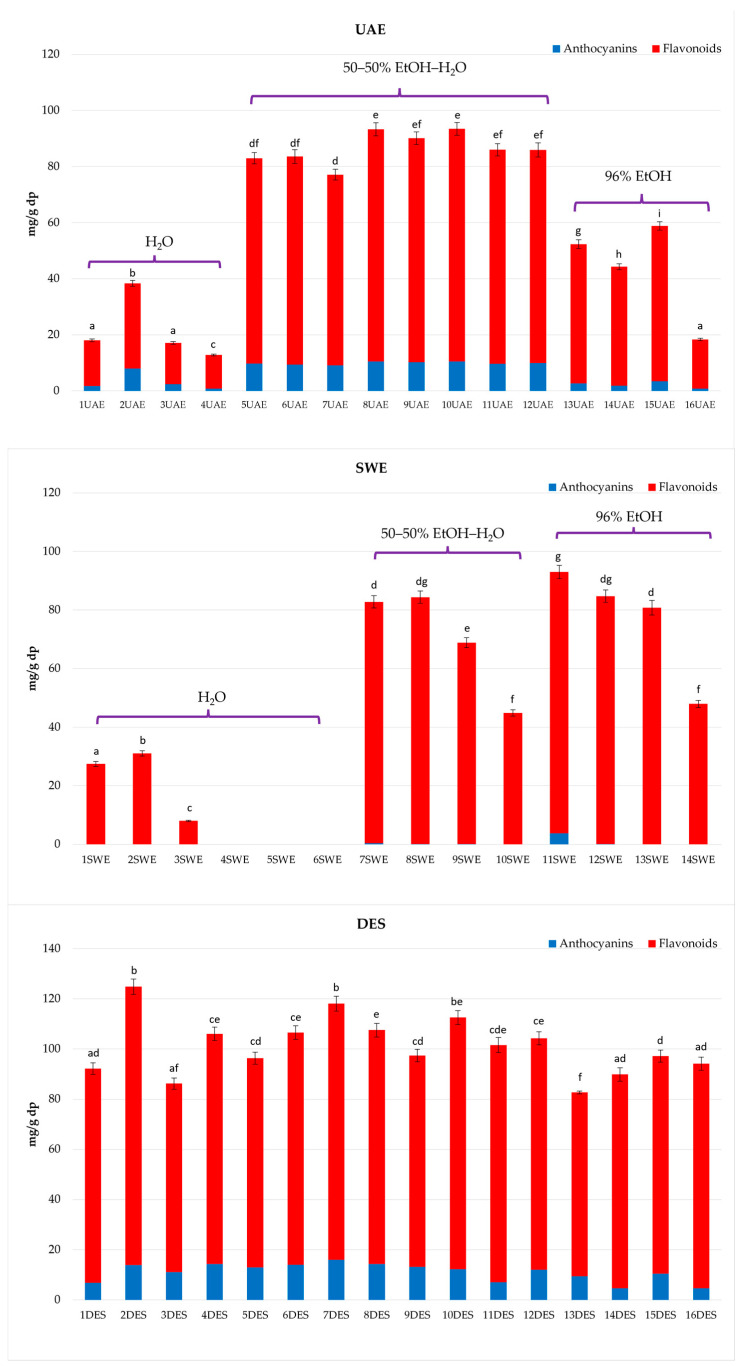
Quantification of phenolic compounds by the LC-PDA method (mg/100 g dp) in *C. sativus* floral by-product extracts. Data are given as mean ± standard deviation (*n* = 3). Mean values within a line with different letters (a–f) are significantly different (homogenous groups) at *p* ≤ 0.05.

Figure 2 shows the total amounts of anthocyanins and flavonoids in the three types of extracts, and Table S1a–c reports the quantification of phenolic compounds by the LC-PDA method (amount expressed as mg/g of dry plant, dp). The comparison of the data obtained from the three different GETs highlights how they can influence the extraction and how they can be selective for a specific class or a single phenolic compound.

Regarding the two most abundant compounds, UAE, SWE, and DESE showed an average value of 72.47%, 77.20%, and 72.70% for delphinidin 3,5-di-*O*-glucoside and 72.62%, 56.80%, and 62.0%, respectively, for kaempferol 3-*O*-sophoroside. 

#### 2.2.1. Ultrasound-Assisted Extraction (UAE) with Sonotrode

Extracts with 100% H_2_O and 96% EtOH as solvents were set at different values of the chosen process parameters according to the response surface methodology (RSM) and applied Box–Behnken design (BBD) [47]. UAE extracts showed different behaviors in the amount of the selected phenolic compounds evaluated according to the applied extraction parameters (Table S1a, Figure 1). In particular, run 10UAE (Figure 1), with EtOH:H_2_O (50:50, *v*/*v)* and both amplitude and impulse set at 60, was the one with the most significant amount of total phenols content (TPC), total flavonoids content (TFC), and anthocyanins content (TAC) (93.43 ± 4.67 mg/g dp, 82.93 ± 4.14 mg/g dp, and 10.50 ± 0.52 mg/g dp, respectively), higher than the extract obtained with the two other solvents but comparable with other extracts obtained with the same solvent (runs 5UAE, 6UAE, 7UAE, 8UAE, 9UAE, 11UAE, and 12UAE). Furthermore, run 10UAE was the one with the highest amounts of delphinidin 3,5-di-*O*-glucoside (8.30 ± 0.42 mg/g dp) and kaempferol 3-*O*-sophoroside (57.28 ± 3.43 mg/g dp). The lowest amount in phenols is represented by the extracts obtained with 100% water (1UAE, 2UAE, 3UAE, 4UAE), particularly 4UAE, with a TPC of 12.80 ± 0.64 mg/g dp, a TFC of 11.98 ± 0.60 mg/g dp, and a TAC of 0.82 ± 0.04 mg/g dp. Interestingly, run 16UAE, with 96% EtOH, also showed one of the lowest amounts of TPC (18.34 ± 0.92 mg/g dp), TFC (17.50 ± 0.88 mg/g dp), and TAC (0.85 ± 0.04 mg/g dp). In this case, amplitude and impulse were set at 20 and 60, respectively, such as in the case of run 4UAE. 

BBD was used to optimize the most important operating variables of the UAE using sonotrode (solvent type, amplitude, impulse) in order to achieve the highest amount of the most abundant detected compounds (delphinidin 3,5-di-*O*-glucoside and kaempferol 3-*O*-sophoroside). The coefficients and the corresponding *p*-values for each investigated response are given in Table S2. The regression coefficients were determined by using multiple linear regression. The degree of statistical significance of each factor is represented with the *p*-value. From the obtained results, it is evident that the solvent type can influence the extraction performance and, finally, the extracts with obtained targeted compounds (Figures S1 and S2). The quadratic term of solvent exhibited the most statistically significant influence on both investigated responses (*p* < 0.0001). These results suggest that the solvent EtOH:H_2_O (50:50, *v*/*v)* is the best choice, followed by 96% EtOH. The linear term of amplitude showed a significant influence only on delphinidin 3,5-di-*O*-glucoside (*p* = 0.0235). The interaction between the input variables was not significant at all (*p* ≥ 0.05).

The statistical significance of regression equations for each selected response was evaluated by analysis of variance (ANOVA) and is given in Table S3. The regression models for all investigated responses were highly significant according to the *p*-value, with satisfactory coefficients of determination (R^2^) (0.9252 and 0.9547). The non-significant lack-of-fit (*p* > 0.05) for each response highlights that the second-order polynomial model is adequate and could be used for the precision of experimental values.

By reviewing the literature, UAE is one of the most common extraction techniques for phenolic compounds in saffron floral by-products [20,22,48,49,50]. Although there are no studies with parameters of amplitude, impulse, and time comparable to those used, the trend is similar to the one highlighted by Turcov et al. [21] that reported, with conventional UAE and EtOH:H_2_O (50:50, *v*/*v)* as the solvent in a liquid/solid ratio of 1:16, one of the highest amounts of TFC. However, we obtained the highest TFC value with EtOH:H_2_O (50:50, *v*/*v*) (82.93 ± 4.14 mg/g dp), while the highest TFC reported by this study (195.61 mg/g quercetin equivalent) was obtained with EtOH:H_2_O (30:70, *v*/*v*). In the case of anthocyanins, the highest TAC was obtained in run 10UAE, which has a similar trend to the results reported by Da Porto et al. [24]. They investigated UAE with sonotrode using EtOH:H_2_O (50:50, *v*/*v)* as a solvent and a solid/liquid ratio of 1:30, showing a TPC of 4971 ± 84 mg gallic acid equivalent/100 g DM and TAC of 527 ± 5 mg cyanidin-glucoside/100 g DM. They confirmed UAE with sonotrode using EtOH:H_2_O (50:50, *v*/*v)* as a new, promising extraction technique for anthocyanins compared with other extraction methods such as conventional solid/liquid extraction and microwave-assisted extraction. 

#### 2.2.2. Subcritical Water Extraction (SWE)

SWE extraction was performed at six different, increasing temperatures using 100% water and at four increasing temperatures using EtOH:H_2_O (50:50, *v*/*v)* and 96% EtOH as solvents. The SWE extracts showed the lowest amounts of anthocyanins; this can be explained by anthocyanin degradation, because these compounds are thermolabile, especially during SWE where high temperatures are used [51]. In fact, in some runs with temperatures set at 175 °C and 200 °C, such as 3SWE, 4SWE, 10SWE, and 14SWE, this class of compounds was completely absent. Furthermore, it was reported that using solvent combinations improved the solubility and increased the interaction between the targeted analyte and the extraction solvent, thereby enhancing the extraction yields. To increase extraction efficiency, a solvent mixture may be used during the extraction process [52]. For this purpose, the addition of EtOH as a solvent results in the higher amount of total phenols even at high temperatures. In particular, run 11SWE was the most interesting for TFC (89.25 ± 4.46 mg/g dp) and TAC (3.72 ± 0.19 mg/g dp), with an amount of total phenols of 92.96 ± 4.65 mg/g dp. In general, the ones with the lowest temperatures showed higher TPCs, especially for flavonoids, and the lower one is shown in the extracts with water as a solvent (1SWE, 2SWE, 3SWE, 4SWE). For instance, 2SWE (Figure 1) showed one of the highest TPCs among all water extracts, but compared with the other solvents at the same temperature, it shows the lower one, while the highest is the one extracted with 96% EtOH (11SWE). Kaempferol-3-*O*-sophoroside is constantly present in the extracts, especially in 11SWE, with an amount of 61.01 ± 3.05 mg/g dp (Table S1b). Interestingly, in run 10SWE (Figure 1), its concentration decreases as the temperature increases, and this could suggest degradation of kaempferol-3-*O*-sophoroside with release of kaempferol aglycone, which was found in higher concentration. Concerning this extraction technique, the use of solvents other than water, such as EtOH:H_2_O (50:50, *v*/*v)* and 96% EtOH, is reported in the literature. Taking water as the solvent, Ahmadian-Kouchaksaraie et al. [27] found that the optimal conditions for the extraction of phenolic compounds were reached at 159 °C for 54 min, which is in line with our results for 2SWE at 150 °C, as the sample with the highest amount of TPC (31.08 ± 1.86 mg/g dp) among all the samples extracted with water. As can be seen in Figure 2, the TFC increases from 1SWE to 2SWE (125 to 150 °C). In this regard, Ahmadian-Kouchaksaraie et al. [27] highlighted that the TFC increases from 120 to 160 °C, pointing out that the temperature of extraction has a major impact on SWE efficiency and that a higher temperature makes flavanol more soluble and influences the saffron-petal cell-wall matrix’s hydrolysis reaction by raising the ionization constant of water. Going forward with the increase in temperature over 150 °C, the flavonoids decrease due to the degradation of phenolic compounds [53]. Anthocyanins are too thermolabile to resist such high temperatures [51], but the use of ethanol allowed a higher amount of anthocyanins. 11SWE is also the extract with the highest amount of delphinidin 3,5-di-*O*-glucoside (2.42 ± 0.12 mg/g dp). The TPC of 11SWE can be compared with 1SWE, which is significantly lower, with an amount of 27.52 ± 1.65 mg/g dp, and 7SWE, which showed an amount of 82.73 ± 4.20 mg/g dp; so, even with the same temperature but different solvents, they are statistically different. Therefore, subcritical water extraction, compared with the one that uses EtOH:H_2_O (50:50, *v*/*v)* and 96% EtOH, resulted in the lowest amount of flavonoids.

#### 2.2.3. Deep Eutectic Solvents Extraction (DESE)

The DES extracts showed similar behaviors among all 16 tested solvents (Table S1c). The one with the highest TAC is 7DES, with an amount of 16.00 ± 0.80 mg/g dp. The highest TFC and TPC is 2DES, with amounts of 110.95 ± 5.55 mg/g dp and 124.86 ± 6.24 mg/g dp, respectively. The lowest amount of total phenols was represented by 13DES (82.69 ± 1.13 mg/g dp), followed by 3DES (86.22 ± 4.31 mg/g dp), both solvents with a high viscosity. Interestingly, this technique showed the highest expression of anthocyanins, pointing out in the chromatograms further peaks at 520 nm, as shown in 14DES (Figure 1). This can be noticed especially for the extracts obtained with ChCl and organic acids. Previous studies showed that NaDESs can be used in conjunction with UAE to extract SFBPs. By comparing additional factors like power, time, and solvent percentage, these studies, like the one we conducted, demonstrate that using NaDESs to extract SFBPs can result in greater TPC values than employing UAE alone [32]. Although there are no previous studies that used the same DES extraction techniques, Lakka et al. [31] used an NaDES composed of L-lactic acid (HBD) like our sample 15DES, but they used glycine as the HBA instead of choline chloride. This solvent was used for the extraction of both flavonoids (TFC: 45.72 mg/g dm) and anthocyanins (TAC: 8.06 mg/g dm), confirming that among flavonoids, the most abundant is kaempferol 3-*O*-sophoroside, with 36.43 ± 2.55 mg/g dm, and among anthocyanins, the most abundant is delphinidin 3,5-di-*O*-glucoside, with 6.28 ± 0.44 mg/g dm. Our investigation showed a similar trend of TFC and TAC; the highest TFC was observed in 2DES, where kaempferol 3-*O*-sophoroside was the most copious (76.95 ± 3.85 mg/g dp), and the highest TAC was observed in 7DES, where delphinidin 3,5-di-*O*-glucoside was the most abundant (12.35 ± 0.62 mg/g dp). Overall, DESs have proven to be an auspicious extraction method for both anthocyanins and flavonoids. The lowest TAC was observed in 16DES, with 4.58 ± 0.32 mg/g dp, and the lowest TFC was noticed in 13DES, with 73.25 ± 3.66 mg/g dp. Moreover, several studies demonstrate how various DESs can be employed as is by the pharmaceutical, cosmetic, and food sectors using, for example, solvents similar to the ones we used, such as lactic acid, glycerol, or urea [54,55].

## 3. Materials and Methods

### 3.1. Chemicals

All the chemicals were of analytical grade. The solvents used for the extraction were purchased from J.T. Baker (Radnor, PA, USA). Methanol and 85% *w*/*w* phosphoric acid were purchased from Sigma-Aldrich (Steinheim, Germany). LC-MS grade acetonitrile, formic acid, and H_2_O were purchased from Merck (Darmastadt, Germany). Isorhamnetin 3-*O*-glucoside, kaempferol, kaempferol 3-*O*-sophoroside, quercetin, quercetin 3-*O*-glucoside, delphinidin 3-*O*-glucoside, delphinidin 3,5-di-*O*-glucoside, petunidin 3-*O*-glucoside, and petunidin 3,5-di-*O*-glucoside were purchased from Extrasynthese (Genay Cedex, France) and TransMIT (Giessen, Germany). Ultrapure water (18 MΩ·cm) was obtained with a Milli-Q Advantage A10 System (Millipore, Milan, Italy).

### 3.2. Plant Material

*Crocus sativus* flower by-products obtained after stigma removal were collected in November 2022 in Sant’Anna Arresi and Turri (Sardinia, Italy). The specimens were identified by Prof. Andrea Maxia (University of Cagliari, Cagliari, Italy), and voucher samples (number DISVA.ALI.07.2022, DISVA.ALI.08.2022) were deposited at the Department of Life and Environmental Sciences of the University of Cagliari (Italy). After the collection, the flowers were cleaned and dried at 45 °C for 24 h (Hendi Dehydrator Profi Line, De Klomp, The Netherlands). Before extraction, the dried floral by-product was homogenized and ground using a standard laboratory miller to obtain a powder sample. The dry plant (dp) was evaluated in triplicate by drying 10 g of floral by-product for 5 h in a thermostatic oven at 105 ± 1 °C to a constant weight.

### 3.3. Extraction Techniques

#### 3.3.1. Ultrasound-Assisted Extraction (UAE) with Sonotrode

For UAE, an ultrasonic probe (UP400St, Hielscher Ultrasonics GmbH, Teltow, Germany) was used with a minimum power of 400 W and a minimum frequency of 24 kHz. The operating conditions for the ultrasound-assisted extraction are shown in Table 1 according to RSM and the applied Box–Behnken design [47]. Independent variables in the design were: solvent (X_1_), amplitude (X_2_), and impulse (X_3_). Design-Expert^®^ commercial software (ver. 9, Stat-Ease Inc., Minneapolis, MN, USA) was used for data analysis. The analysis of variance (ANOVA) was also used to evaluate the quality of the fitted model, and the test of statistical difference was based on the total error criteria with a confidence level of 95.0%.

Briefly, 1 g of powdered sample was placed in 30 mL of three different solvents (100% water, 50:50 *v*/*v* EtOH:H_2_O, and 96:4 *v*/*v* EtOH:H_2_O) depending on BBD, while extraction time was constant (3 min) during the experiment. The obtained extracts were filtered through a PTFE 0.45 μm filter before further analyses. 

#### 3.3.2. Subcritical Water Extraction (SWE)

The extraction was carried out in a handmade subcritical water extraction system described in detail by Jokić et al. [37]. The powdered sample (10 g/100 mL) was placed into a 500 mL extraction vessel made from stainless steel (AISI 304). The extractions were performed at four different temperatures (125 °C, 150 °C, 175 °C, and 200 °C) for the two solvents 50:50% and 96:4 EtOH:H_2_O % *v*/*v*, and six different temperatures (125 °C, 150 °C, 175 °C, 200 °C, 220 °C, and 250 °C) for distilled H_2_O, with a reaction time of 20 min at a working pressure of 40 bar. The sample–water mixture was poured into the reactor. The extraction vessel was heated in an oven to the desired temperature (125–250 °C). The mixture was stirred with a magnetic stirrer placed below the extractor vessel to obtain adequate stirring of water and material. N_2_ was used to control pressure and provide an inert state during the extractions. When the extraction was finished, the reactor was rapidly cooled in an ice bath. The reactor content was filtered through filter paper, and the water extracts were obtained.

#### 3.3.3. Extraction with Deep Eutectic Solvents (DESs)

For the DES extraction, the method outlined by Kovač et al. [38], with several modifications, was used. DES mixtures were prepared considering different ratios of HBD/HBA to prepare the eutectic mixture: choline chloride (HBA) was always used at 5 g, 16 different HBDs were chosen, and their amounts were calculated referring to their molecular weights and ratios (Table 1). The solvents were heated until they formed a clear liquid, and then they were cooled down. In appropriate vials, 1 g of glass beads with 15 mg of matrix were weighed on an analytical scale. Subsequently, 800 μL of solvents were added in the vials together with 200 μL of H_2_O Milli Q. All of the samples were performed in triplicate. The vials were put in a Bead Ruptor 12 (Omni International), with speed set at 4.00 m/s, time set at 2.00 min, and 2 cycles of homogenization. The vials were centrifuged for 5 min before collecting the samples into 1 mL Eppendorf tubes. 

### 3.4. High-Resolution HPLC-ESI-QToF-MS/MS and HPLC-DAD Analysis

For the qualitative and quantitative assessment of the saffron floral by-products, the method described by De Luca et al. [56] was used. Briefly, the analytical setup included an advanced ion mobility QToF LC/MS system equipped with a 1290 Infinity II UPLC and a 6560 IM-QToF (Agilent Technologies Inc., Palo Alto, CA, USA), and experiments were conducted using an electrospray ionization (ESI) source set to operate in positive and negative ion modes. ESI/QToF MS data were then analyzed using the MassHunter Workstation Qualitative Analysis software v. 10.0 (Agilent Technologies). The MassHunter METLIN metabolite PCDLdatabase v. B.08.00 (Agilent Technologies) and Sirius^®^ software v. 4.7.4 were used for the tentative identification of the metabolites and to predict fragmentation and molecular formulae [41,57]. Experimental MS/MS spectra were further compared with fragmentation patterns reported in the literature or with spectra reported in a public repository of mass spectral data [42]. The quantitative analysis of targeted phenolic compounds was performed using a 1260 Infinity II HPLC system equipped with a G4212B photodiode array detector (Agilent Technologies). The chromatograms and spectra were processed using OpenLab CDS software v. 2.5 (Agilent Technologies), and phenolic compounds were detected and quantified based on absorption at characteristic wavelengths (anthocyanins at 520 nm and flavonols at 360 nm). The calibration curves were built by correlating the peak area with the concentration by the least squares method, with R^2^ > 0.999 in a 0.2–10.0 mg/L range for all the standards. For the analysis, the extracts were dissolved with MeOH (1:50 *w*/*v* extract/solvent ratio) and diluted 1:10 *v*/*v* for the UAE and DES samples and 1:20 for the SWE samples with 0.22 M phosphoric acid. The solutions were filtered with a 0.22 μm CA syringe filter before injection.

## 4. Conclusions

The deep investigation by (HR) LC-ESI-QTOF MS/MS analysis of the composition of the SFBPs extracted with the three GETs allowed us to assess differences in the extraction of phenolic compounds. Among the variable extraction parameters, RSM and BBD have shown that, between UAE, the solvent was the most decisive for the extraction of anthocyanins and flavonoids, especially for delphinidin 3,5-di-*O*-glucoside and kaempferol 3-*O*-sophoroside, the most abundant detected compounds. Indeed, water was the solvent that allowed the least efficient extraction for both UAE and SWE. Interestingly, EtOH:H_2_O (50:50, *v*/*v*) was the best solvent for UAE, and 96% EtOH was the best one for SWE. In SWE, temperature also played a fundamental role, which was shown to be optimal at 125 and 150 °C, a temperature above which there is a notable loss of TPC and TFC. It is interesting to note that in the case of water and EtOH:H_2_O (50:50, *v*/*v*), the highest TPC is observed at 150 °C, while in the case of 96% EtOH, the highest TPC and TFC are at 125 °C. Moreover, all 16 DESs showed the highest amounts for both classes of compounds. Comparing the three GETs, DESE with ChCl:butane-1,4-diol was the finest for the extraction of anthocyanins (7DES). It was also the one with the highest amount of delphinidin 3,5 di-*O*-glucoside, the most representative among the anthocyanins. Among the flavonoids, the most representative was kaempferol 3-*O*-sophoroside, and it was the most abundant in 2DES (ChCl:N-methylurea as the solvent). In terms of optimal extraction, DESE was followed by UAE, SWE (50:50% *v*/*v* EtOH:H_2_O and 96% EtOH, respectively), and then SWE with water, which showed lower amounts of phenolic compounds when compared with other extraction techniques, especially regarding anthocyanins. Depending on how the extract will be used, more research is required to determine which DES solvents are optimal. Given that the extraction conditions for the different GETs were different, further investigations would be useful to fully understand the behavior of the solvents in each extraction technique, comparing similar temperature, extraction time, and solvent/plant material ratio. As for DES, additional studies can help understand how to remove the solvent and exploit the extract to its full potential. Finally, GETs are an environmentally friendly method for obtaining highly biologically interesting chemicals from SFBPs that have potential use in the food, pharmaceutical, nutraceutical, and cosmetic industries. They also serve as a valuable tool for reducing waste from the agri-food sector.

## Figures and Tables

**Table 1:** *C. sativus* flower by-product samples and parameters of the green extraction techniques used.

Sample Code *	Extraction Parameters		
	Amplitude	Impulse	Solvent
1UAE	100	60	100% H_2_O
2UAE	60	20	
3UAE	60	100	
4UAE	20	60	
5UAE	100	100	EtOH:H_2_O (50:50, *v*/*v)*
6UAE	100	20	
7UAE	60	60	
8UAE	60	60	
9UAE	60	60	
10UAE	60	60	
11UAE	20	100	
12UAE	20	20	
13UAE	100	60	96% EtOH
14UAE	60	20	
15UAE	60	100	
16UAE	20	60	
	Temperature (°C)	Solvent	
1SWE	125	100% H_2_O	
2SWE	150		
3SWE	175		
4SWE	200		
5SWE	225		
6SWE	250		
7SWE	125	EtOH:H_2_O (50:50, *v*/*v)*	
8SWE	150		
9SWE	175		
10SWE	200		
11SWE	125	96% EtOH	
12SWE	150		
13SWE	175		
14SWE	200		
	Extraction solvent		
1DES	Choline chloride:urea 1:2–H_2_O content (80–20, *v*,*v*)		
2DES	Choline chloride:*N*-methyl urea 1:3–H_2_O (80–20, *v*,*v*)		
3DES	Choline chloride:thiourea 1:2–H_2_O (80–20, *v*,*v*)		
4DES	Choline chloride:xylitol 1:1–H_2_O (80–20, *v*,*v*)		
5DES	Choline chloride:sorbitol 1:1–H_2_O (80–20, *v*,*v*)		
6DES	Choline chloride:acetamide 1:2–H_2_O (80–20, *v*,*v*)		
7DES	Choline chloride:butane-1,4-diol 1:2–H_2_O (80–20, *v*,*v*)		
8DES	Choline chloride:ethane-1,2-diol 1:2–H_2_O (80–20, *v*,*v*)		
9DES	Choline chloride:glycerol 1:2–H_2_O (80–20, *v*,*v*)		
10DES	Choline chloride:oxalic acid 1:1–H_2_O (80–20, *v*,*v*)		
11DES	Choline chloride:1,3-dimethylurea 1:2–H_2_O (80–20, *v*,*v*)		
12DES	Choline chloride:maleic acid 1:1–H_2_O (80–20, *v*,*v*)		
13DES	Choline chloride:malic acid 1:1–H_2_O (80–20, *v*,*v*)		
14DES	Choline chloride:malonic acid 1:1–H_2_O (80–20, *v*,*v*)		
15DES	Choline chloride:lactic acid 1:2–H_2_O (80–20, *v*,*v*)		
16DES	Choline chloride:levulinic 1:2–H_2_O (80–20, *v*,*v*)		

* Extraction technique: UAE, ultrasound-assisted extraction; SWE, subcritical water extraction; DES, deep eutectic solvent.

## Data Availability

Data are contained within the article and Supplementary Materials.

## Supplementary Materials

The following supporting information can be downloaded at: https://www.mdpi.com/article/10.3390/molecules29112600/s1, Table S1: Quantification of phenolic compounds by the LC-PDA method (mg/g dp); Table S2: Regression coefficient of polynomial function of the most significant response surfaces during UAE; Table S3: Analysis of variance (ANOVA) of the selected modeled responses during UAE; Figure S1: Three-dimensional plots for obtained delphinidin 3,5-di-*O*-glucoside in extracts as a function of UAE process parameters; Figure S2: Three-dimensional plots for obtained kaempferol 3-*O*-sophoroside in extracts as a function of UAE process parameters.

## References

[1] Shahnoushi N., Abolhassani L., Kavakebi V., Reed M., Saghaian S. (2020). Chapter 21—Economic analysis of saffron production. Saffron: Science, Technology and Health.

[2] Cerdá-Bernad D., Clemente-Villalba J., Valero-Cases E., Pastor J.-J., Frutos M.-J. (2022). Novel insight into the volatile profile and antioxidant properties of *Crocus sativus* L. flowers. Antioxidants.

[3] Comunian T.A., Silva M.P., Souza C.J.F. (2021). The use of food by-products as a novel for functional foods: Their use as ingredients and for the encapsulation process. Food Sci. Technol..

[4] Cerdá-Bernad D., Valero-Cases E., Pérez-Llamas F., Pastor J.J., Frutos M.J. (2023). Underutilized *Crocus Sativus* L. flowers: A hidden source of sustainable high value-added ingredients. Plant Foods Hum. Nutr..

[5] Goupy P., Vian M.A., Chemat F., Caris-Veyrat C. (2013). Identification and quantification of flavonols, anthocyanins and lutein diesters in tepals of *Crocus sativus* by ultra-performance liquid chromatography coupled to diode array and ion trap mass spectrometry detections. Ind. Crops Prod..

[6] Tuberoso C.I.G., Rosa A., Montoro P., Fenu M.A., Pizza C. (2016). Antioxidant activity, cytotoxic activity and metabolic profiling of juices obtained from saffron (*Crocus sativus* L.) floral by-products. Food Chem..

[7] Campos-Vidal Y., Zamilpa A., Jiménez-Ferrer E., Jiménez-Aparicio A.R., Camacho-Díaz B.H., Trejo-Tapia G., Tapia-Maruri D., Monterrosas-Brisson N., Herrera-Ruiz M. (2022). A mixture of kaempferol-3-*O*-sambubioside and kaempferol-3-*O*-sophoroside from *Malvaviscus arboreus* prevents ethanol-induced gastric inflammation, oxidative stress, and histologic changes. Plants.

[8] Yang L., He J. (2022). Anti-inflammatory effects of flavonoids and phenylethanoid glycosides from *Hosta plantaginea* flowers in LPS-stimulated RAW 264.7 macrophages through inhibition of the NF-κB signaling pathway. BMC Complement. Med. Ther..

[9] Ye H., Luo J., Hu D., Yang S., Zhang A., Qiu Y., Ma X., Wang J., Hou J., Bai J. (2021). Total flavonoids of *Crocus sativus* petals release tert-butyl hydroperoxide-induced oxidative stress in BRL-3A cells. Oxid. Med. Cell. Longev..

[10] Maestre-Hernández A.-B., Vicente-López J.-J., Pérez-Llamas F., Candela-Castillo M.-E., García-Conesa M.-T., Frutos M.-J., Cano A., Hernández-Ruiz J., Arnao M.B. (2023). Antioxidant activity, total phenolic and flavonoid contents in floral saffron bio-residues. Processes.

[11] Wali A.F., Abou Alchamat H.A., Hariri H.K., Hariri B.K., Menezes G.A., Zehra U., Rehman M.U., Parvaiz A. (2020). Antioxidant, antimicrobial, antidiabetic and cytotoxic activity of *Crocus sativus* L. petals. Appl. Sci..

[12] Khazaei K.M., Jafari S.M., Ghorbani M., Hemmati Kakhki A., Sarfarazi M. (2016). Optimization of anthocyanin extraction from saffron petals with response surface methodology. Food Anal. Methods.

[13] Righi V., Parenti F., Tugnoli V., Schenetti L., Mucci A. (2015). *Crocus sativus* petals: Waste or valuable resource? The answer of high-resolution and high-resolution magic angle spinning nuclear magnetic resonance. J. Agric. Food Chem..

[14] Sánchez-Vioque R., Santana-Méridas O., Polissiou M., Vioque J., Astraka K., Alaiz M., Herraiz-Peñalver D., Tarantilis P.A., Girón-Calle J. (2016). Polyphenol composition and in vitro antiproliferative effect of corm, tepal and leaf from *Crocus sativus* L. on human colon adenocarcinoma cells (Caco-2). J. Funct. Foods.

[15] Orabona C., Orecchini E., Volpi C., Bacaloni F., Panfili E., Pagano C., Perioli L., Belladonna M.L. (2022). *Crocus sativus* L. petal extract inhibits inflammation and osteoclastogenesis in RAW 264.7 cell model. Pharmaceutics.

[16] Jadouali S.M., Atifi H., Mamouni R., Majourhat K., Bouzoubaâ Z., Laknifli A., Faouzi A. (2019). Chemical characterization and antioxidant compounds of flower parts of Moroccan *Crocus sativus* L.. J. Saudi Soc. Agric. Sci..

[17] Álvarez A., Terreros S., Cocero M.J., Mato R.B. (2021). Microwave pretreatment for the extraction of anthocyanins from saffron flowers: Assessment of product quality. Antioxidants.

[18] Cerdá-Bernad D., Baixinho J.P., Fernández N., Frutos M.J. (2022). Evaluation of microwave-assisted extraction as a potential green technology for the isolation of bioactive compounds from saffron (*Crocus sativus* L.) floral by-products. Foods.

[19] Gil K.A., Tuberoso C.I.G. (2021). Crucial challenges in the development of green extraction technologies to obtain antioxidant bioactive compounds from agro-industrial by-products. Chem. Biochem. Eng. Q..

[20] Mottaghipisheh J., Mahmoodi Sourestani M., Kiss T., Horváth A., Tóth B., Ayanmanesh M., Khamushi A., Csupor D. (2020). Comprehensive chemotaxonomic analysis of saffron crocus tepal and stamen samples, as raw materials with potential antidepressant activity. J. Pharm. Biomed. Anal..

[21] Turcov D., Barna A.S., Apreutesei O.T., Trifan A., Puitel A.C., Suteu D. (2022). Valorization of bioactive compounds from residual saffron biomass (*Crocus sativus* L.) to obtain high value added dermato-cosmetic products. BioResources.

[22] Stelluti S., Caser M., Demasi S., Scariot V. (2021). Sustainable processing of floral bio-residues of saffron (*Crocus sativus* L.) for valuable biorefinery products. Plants.

[23] Hashemi Gahruie H., Parastouei K., Mokhtarian M., Rostami H., Niakousari M., Mohsenpour Z. (2020). Application of innovative processing methods for the extraction of bioactive compounds from saffron (*Crocus sativus*) petals. J. Appl. Res. Med. Aromat. Plants.

[24] Da Porto C., Natolino A. (2018). Extraction kinetic modelling of total polyphenols and total anthocyanins from saffron floral bio-residues: Comparison of extraction methods. Food Chem..

[25] Cheng Y., Xue F., Yu S., Du S., Yang Y. (2021). Subcritical water extraction of natural products. Molecules.

[26] Vardakas A., Vassilev K., Nenov N., Passon M., Shikov V., Schieber A., Mihalev K. (2023). Combining enzymatic and subcritical water treatments for green extraction of polyphenolic co-pigments from saffron tepals. Waste Biomass Valorization.

[27] Ahmadian-Kouchaksaraie Z., Niazmand R., Najafi M.N. (2016). Optimization of the subcritical water extraction of phenolic antioxidants from *Crocus sativus* petals of saffron industry residues: Box-Behnken design and principal component analysis. Innov. Food Sci. Emerg. Technol..

[28] Pappas V.M., Athanasiadis V., Palaiogiannis D., Poulianiti K., Bozinou E., Lalas S.I., Makris D.P. (2021). Pressurized liquid extraction of polyphenols and anthocyanins from saffron processing waste with aqueous organic acid solutions: Comparison with stirred-tank and ultrasound-assisted techniques. Sustainability.

[29] Ruesgas-Ramón M., Figueroa-Espinoza M.C., Durand E. (2017). Application of Deep Eutectic Solvents (DES) for phenolic compounds extraction: Overview, challenges, and opportunities. J. Agric. Food Chem..

[30] SShikov A.N., Obluchinskaya E.D., Flisyuk E.V., Terninko I.I., Generalova Y.E., Pozharitskaya O.N. (2022). The impact of natural deep eutectic solvents and extraction method on the co-extraction of trace metals from *Fucus vesiculosus*. Mar. Drugs.

[31] Lakka A., Grigorakis S., Karageorgou I., Batra G., Kaltsa O., Bozinou E., Lalas S., Makris D.P. (2019). Saffron processing wastes as a bioresource of high-value added compounds: Development of a green extraction process for polyphenol recovery using a natural deep eutectic solvent. Antioxidants.

[32] Cerdá-Bernad D., Pitterou I., Tzani A., Detsi A., Frutos M.J. (2023). Novel chitosan/alginate hydrogels as carriers of ohenolic-enriched extracts from saffron floral by-products using natural deep eutectic solvents as green extraction media. Food Sci..

[33] Sitthisak C., Nisoa M., Chunglok W., Prasopthum A., Phaisan S., Putalun W., Kanchanapoom T., Juengwatanatrakul T., Yusakul G. (2024). Efficient extraction of quassinoids and alkaloids from *Eurycoma longifolia* Jack roots using natural deep eutectic solvents and microwave-assisted extraction. Microchem. J..

[34] Petrochenko A.A., Orlova A., Frolova N., Serebryakov E.B., Soboleva A., Flisyuk E.V., Frolov A., Shikov A.N. (2023). Natural deep eutectic solvents for the extraction of triterpene saponins from *Aralia elata* var. *mandshurica* (Rupr. & Maxim.). J. Wen. Molecules.

[35] Yao J., Zeng J., Tang H., Cheng Y., Tan J., Li T., Li X., He J., Zhang Y. (2023). Effect of deep eutectic solvent extraction on *Auricularia auricula* polysaccharide solubilization and antioxidant potential. Sustain. Chem. Pharm..

[36] Obluchinskaya E.D., Pozharitskaya O.N., Shevyrin V.A., Kovaleva E.G., Flisyuk E.V., Shikov A.N. (2023). Optimization of extraction of phlorotannins from the arctic *Fucus vesiculosus* using natural deep eutectic solvents and their HPLC profiling with tandem high-resolution mass spectrometry. Mar. Drugs.

[37] Jokić S., Aladić K., Šubarić D. (2018). Subcritical water extraction laboratory plant design and application. Annu. Croat. Acad. Eng..

[38] Kovač M.J., Jokić S., Jerković I., Molnar M. (2022). Optimization of deep eutectic solvent extraction of phenolic acids and tannins from *Alchemilla vulgaris* L.. Plants.

[39] Gil K.A., Jokić S., Cikoš A.-M., Banožić M., Jakovljević Kovač M., Fais A., Tuberoso C.I.G. (2023). Comparison of different green extraction techniques used for the extraction of targeted flavonoids from edible feijoa (*Acca sellowiana* (O.Berg) Burret) flowers. Plants.

[40] Blaženović I., Kind T., Ji J., Fiehn O. (2018). Software tools and approaches for compound identification of LC-MS/MS Data in metabolomics. Metabolites.

[41] Hoffmann M.A., Nothias L.F., Ludwig M., Fleischauer M., Gentry E.C., Witting M., Dorrestein P.C., Dührkop K., Böcker S. (2022). High-confidence structural annotation of metabolites absent from spectral libraries. Nat. Biotechnol..

[42] KNApSAcK Core System. http://www.knapsackfamily.com/knapsack_core/top.php.

[43] Chen N., Xiang J., Liu Y., Li H., Yang B. (2021). Preparation and characterization of antioxidant flavonoid-enriched extract from saffron by-product: A combination of ultrasound-assisted extraction and macroporous resin purification. Chem. Papers.

[44] Serrano-Díaz J., Sánchez A.M., Martínez-Tomé M., Winterhalter P., Alonso G.L. (2014). Flavonoid determination in the quality control of floral bioresidues from *Crocus sativus* L.. J. Agric. Food Chem..

[45] Gavrilova V., Kajdžanoska M., Gjamovski V., Stefova M. (2011). Separation, characterization and quantification of phenolic compounds in blueberries and red and black currants by HPLC-DAD-ESI-MSn. J. Agric. Food Chem..

[46] Giusti M.M., Rodríguez-Saona L.E., Griffin D., Wrolstad R.E. (1999). Electrospray and tandem mass spectroscopy as tools for anthocyanin characterization. J. Agric. Food Chem..

[47] Bas D., Boyaci I.H. (2007). Modeling and optimization I: Usability of response surface methodology. J. Food Eng..

[48] Cusano E., Consonni R., Petrakis E.A., Astraka K., Cagliani L.R., Polissiou M.G. (2018). Integrated analytical methodology to investigate bioactive compounds in *Crocus sativus* L. flowers. Phytochem. Anal..

[49] Serrano-Díaz J., Ana M., Sánchez A.M., Maggi L., Martínez-Tomé M., García-Diz L., Murcia M.A., Alonso G.L. (2012). Increasing the applications of *Crocus sativus* flowers as natural antioxidants. J. Food Sci..

[50] Sun C., Nile S.H., Zhang Y., Qin L., El-Seedi H.R., Daglia M., Kai G. (2020). Novel insight into utilization of flavonoid glycosides and biological properties of saffron (*Crocus sativus* L.) flower byproducts. J. Agric. Food Chem..

[51] Sadilova E., Stintzing F.C., Carle R. (2006). Thermal degradation of acylated and nonacylated anthocyanins. J. Food Sci..

[52] Mustafa A., Turner C. (2011). Pressurized liquid extraction as a green approach in food and herbal plants extraction: A review. Anal. Chim. Acta.

[53] Singh P.P., Saldaña M.D.A. (2011). Subcritical water extraction of phenolic compounds from potato peel. Food Res. Int..

[54] Villa C., Caviglia D., Robustelli della Cuna F.S., Zuccari G., Russo E. (2024). NaDES application in cosmetic and pharmaceutical fields: An overview. Gels.

[55] Morgana N.M., Magdalena E., Fernandez M.d.L.A., Fernanda S.M. (2022). NADES for food industry innovation: Novel bioadditives based on olive oil byproducts. Food Bioprod. Process..

[56] De Luca M., Tuberoso C.I.G., Pons R., García M.T., Morán M.d.C., Ferino G., Vassallo A., Martelli G., Caddeo C. (2023). Phenolic fingerprint, bioactivity and nanoformulation of *Prunus spinosa* L. fruit extract for skin delivery. Pharmaceutics.

[57] Dührkop K., Fleischauer M., Ludwig M., Aksenov A.A., Melnik A.V., Meusel M., Dorrestein P.C., Rousu J., Böcker S. (2019). SIRIUS 4: A rapid tool for turning tandem mass spectra into metabolite structure information. Nat. Methods.

