# Supplementary material for: Exploring Phenolic Compounds Extraction from Saffron (C. sativus) Floral By-Products Using Ultrasound-Assisted Extraction, Deep Eutectic Solvent Extraction, and Subcritical Water Extraction
Source: Molecules. 2024 Jun 1;29(11):2600. doi: 10.3390/molecules29112600 (PMC11173527; doi:10.3390/molecules29112600)
Supplement: Supplementary file 1 [file molecules-29-02600-s001.zip › molecules-3008425-supplementary.pdf]

Supplementary File

Exploring Phenolic Compounds Extraction from Saffron (*C. sativus*) Floral By-Products using Ultrasound-Assisted Extraction, Deep Eutectic Solvent Extraction, and Subcritical Water Extraction

Valentina Masala, Stela Jokić, Krunoslav Aladić, Maja Molnar and Carlo Ignazio Giovanni Tuberoso

Table S1. Quantification of phenolic compounds by LC-PDA method (mg/g dp)

(a)

Compounds §		1UAE	2UAE	3UAE	4UAE	5UAE	6UAE	7UAE	8UAE	9UAE	10UAE	11UAE	12UAE	13UAE	14UAE	15UAE	16UAE
**Anthocyanins**																	
Delphinidin 3,5-di-*O*-glucoside	A1	1.34 ± 0.07a	6.02 ± 0.30b	1.85 ± 0.09c	0.35 ± 0.03d	7.67 ± 0.38ef	7.31 ± 0.37e	7.07 ± 0.35e	8.23 ± 0.41f	8.01 ± 0.40ef	8.30 ± 0.42f	7.60 ± 0.38e	7.62 ± 0.53ef	1.81 ± 0.09c	1.21 ± 0.08a	2.29 ± 0.16g	0.55 ± 0.04h
Petunidin 3,5-di-*O*-glucoside	A2	0.26 ± 0.01a	1.06 ± 0.05b	0.33 ± 0.02c	0.09 ± 0.01d	1.26 ± 0.08e	1.20 ± 0.06e	1.22 ± 0.09e	1.32 ± 0.07e	1.28 ± 0.06e	1.28 ± 0.08e	1.24 ± 0.06e	1.31 ± 0.09e	0.48 ± 0.02f	0.33 ± 0.03c	0.62 ± 0.05g	0.15 ± 0.02h
Delphinidin 3-*O*-glucoside	A3	0.06 ± 0.00a	0.31 ± 0.02b	0.13 ± 0.01c	0.14 ± 0.00c	0.55 ± 0.03d	0.53 ± 0.04d	0.52 ± 0.03d	0.58 ± 0.03d	0.58 ± 0.03d	0.59 ± 0.06d	0.55 ± 0.03d	0.57 ± 0.04d	0.27 ± 0.02b	0.22 ± 0.01e	0.31 ± 0.02b	0.09 ± 0.00f
Petunidin 3-*O*-glucoside	A4	0.02 ± 0.00a	0.07 ± 0.01b	0.03 ± 0.00c	0.05 ± 0.01be	0.10 ± 0.01d	0.10 ± 0.00d	0.10 ± 0.01d	0.10 ± 0.00d	0.10 ± 0.01d	0.10 ± 0.01d	0.10 ± 0.00d	0.11 ± 0.01d	0.06 ± 0.01b	0.05 ± 0.00e	0.07 ± 0.01b	0.02 ± 0.00a
Other anthocyanins[a]		0.07 ± 0.00a	0.58 ± 0.03b	0.08 ± 0.00c	0.20 ± 0.01d	0.23 ± 0.01efg	0.22 ± 0.00e	0.22 ± 0.01eg	0.25 ± 0.01f	0.24 ± 0.02efg	0.23 ± 0.01efg	0.23 ± 0.00g	0.34 ± 0.02h	0.06 ± 0.00i	0.04 ± 0.01j	0.12 ± 0.01k	0.03 ± 0.01j
**Total**		1.75 ± 0.09a	8.04 ± 0.40b	2.41 ± 0.16c	0.82 ± 0.04d	9.80 ± 0.49ef	9.36 ± 0.56e	9.13 ± 0.46e	10.48 ± 0.52f	10.20 ± 0.61ef	10.50 ± 0.52f	9.72 ± 0.48ef	9.95 ± 0.50ef	2.67 ± 0.19c	1.85 ± 0.15a	3.41 ± 0.24g	0.85 ± 0.04d
**Flavonoids**																	
Kaempferol sophoroside-glucoside[b]	1	nd	nd	nd	nd	0.16 ± 0.01a	0.15 ± 0.02ab	0.15 ± 0.01ab	0.17 ± 0.02a	0.16 ± 0.01a	0.13 ± 0.01b	0.15 ± 0.01ab	0.16 ± 0.01a	0.04 ± 0.00c	0.03 ± 0.00d	0.05 ± 0.01c	nd
Kaempferol tri-*O*-glucoside[b]	2	nd	0.08 ± 0.00a	nd	nd	5.97 ± 0.30bc	5.83 ± 0.29bd	5.51 ± 0.23b	6.54 ± 0.33c	6.29 ± 0.31cd	6.58 ± 0.33cd	5.98 ± 0.29bcd	6.03 ± 0.36bcd	2.76 ± 0.14e	2.00 ± 0.10f	3.24 ± 0.16g	0.87 ± 0.04h
Kaempferol tri-*O*-glucoside (acetyl)[b]	3	0.05 ± 0.00a	0.04 ± 0.00b	nd	nd	0.20 ± 0.03c	0.19 ± 0.01c	0.19 ± 0.01c	0.22 ± 0.02c	0.20 ± 0.01c	0.21 ± 0.02c	0.20 ± 0.01c	0.21 ± 0.01c	0.11 ± 0.01d	0.09 ± 0.01d	0.14 ± 0.02de	0.04 ± 0.00b
Quercetin di-*O*-glucoside[c]	5	0.35 ± 0.02a	0.54 ± 0.03b	0.21 ± 0.02c	0.80 ± 0.04d	0.99 ± 0.05e	1.01 ± 0.10ef	0.95 ± 0.05eg	1.13 ± 0.06f	1.10 ± 0.05fg	1.11 ± 0.06fg	1.03 ± 0.05ef	1.04 ± 0.05ef	0.19 ± 0.02c	0.13 ± 0.01h	0.22 ± 0.01c	0.06 ± 0.01i
Quercetin sophoroside[c]	6	1.11 ± 0.06a	1.98 ± 0.12b	0.93 ± 0.05c	nd	4.56 ± 0.23de	4.47 ± 0.22de	4.35 ± 0.23de	4.93 ± 0.25d	4.80 ± 0.34de	4.84 ± 0.29de	4.55 ± 0.23de	4.74 ± 0.24de	2.43 ± 0.12e	1.97 ± 0.10b	2.73 ± 0.14f	0.78 ± 0.04g
Kaempferol 3-*O*-sophoroside	9	12.21 ± 0.61a	21.75 ± 1.09b	11.43 ± 0.57a	9.45 ± 0.47c	50.63 ± 2.53d	51.35 ± 3.08de	47.68 ± 2.37d	57.17 ± 2.86e	55.23 ± 2.76de	57.28 ± 3.43e	52.85 ± 2.64de	52.53 ± 3.15de	37.40 ± 1.87f	32.49 ± 1.62g	41.54 ± 2.49h	13.57 ± 0.68i
Isorhamnetin 3-*O*-glucoside	16	nd	1.05 ± 0.10a	0.37 ± 0.02b	nd	0.14 ± 0.01c	0.16 ± 0.01cd	0.14 ± 0.01c	0.18 ± 0.02de	0.18 ± 0.01de	0.21 ± 0.02e	0.16 ± 0.01cd	0.16 ± 0.02cd	0.06 ± 0.00e	0.05 ± 0.01e	0.07 ± 0.00f	nd
Quercetin	18	0.07 ± 0.00a	0.13 ± 0.01b	0.04 ± 0.01c	0.34 ± 0.02d	0.26 ± 0.02e	0.35 ± 0.02d	0.31 ± 0.03d	0.39 ± 0.02f	0.38 ± 0.02f	0.39 ± 0.02f	0.35 ± 0.03df	0.33 ± 0.02d	0.22 ± 0.01g	0.16 ± 0.02b	0.22 ± 0.02g	0.06 ± 0.00h
Kaempferol	21	0.35 ± 0.03a	0.41 ± 0.02b	0.12 ± 0.01c	1.21 ± 0.06d	1.14 ± 0.07d	1.61 ± 0.11e	0.05 ± 0.03f	1.73 ± 0.09e	1.76 ± 0.11e	1.97 ± 0.09g	1.56 ± 0.12e	1.18 ± 0.10d	1.19 ± 0.09d	0.96 ± 0.05h	0.99 ± 0.05h	0.37 ± 0.02ab
Other flavonoids[b]		2.14 ± 0.11a	4.34 ± 0.23b	1.61 ± 0.08c	0.18 ± 0.01d	9.13 ± 0.46e	9.10 ± 0.55e	8.65 ± 0.43e	10.35 ± 0.52f	9.82 ± 0.49ef	10.20 ± 0.51f	9.45 ± 0.47ef	9.60 ± 0.48ef	5.24 ± 0.26g	4.55 ± 0.23b	6.22 ± 0.31h	1.74 ± 0.09c
**Total**		16.29 ± 0.81a	30.33 ± 1.21b	14.71 ± 0.74c	11.98 ± 0.60d	73.17 ± 3.66e	74.22 ± 3.71e	67.97 ± 3.40e	82.81 ± 4.14f	79.93 ± 3.99ef	82.93 ± 4.14f	76.28 ± 3.81ef	76.00 ± 3.80ef	49.63 ± 2.48g	42.42 ± 2.12h	55.41 ± 2.77i	17.50 ± 0.88a
**TOTAL PHENOLS**		18.04 ± 1.08a	38.37 ± 1.92b	17.12 ± 0.86a	12.80 ± 0.64c	82.98 ± 4.11df	83.57 ± 5.01df	77.11 ± 3.86d	93.29 ± 4.66e	90.14 ± 4.51ef	93.43 ± 4.67e	86.00 ± 4.30ef	85.94 ± 5.15def	52.31 ± 3.13g	44.27 ± 2.21h	58.82 ± 2.94i	18.34 ± 0.92a

§ peak number as reported in Table 2. [a] expressed as delphinidin 3,5-di-*O*-glucoside; [b] expressed as kaempferol 3-*O*-sophoroside equivalents; [c] expressed as quercetin 3-*O*-glucoside equivalents. nd: not detected. Data are given as mean ± standard deviation ($n = 3$). Mean values within a line with different letters are significantly different (homogenous groups) at $p \leq 0.05$.

(b)

Compounds §		1SWE	2SWE	3SWE	4SWE	5SWE	6SWE	7SWE	8SWE	9SWE	10SWE	11SWE	12SWE	13SWE	14SWE
**Anthocyanins**															
Delphinidin 3,5-di-*O*-glucoside	A1	0.04 ± 0.01a	0.02 ± 0.00b	nd	nd	nd	nd	0.27 ± 0.01c	0.08 ± 0.01d	0.06 ± 0.01ad	nd	2.42 ± 0.12e	0.07 ± 0.00d	0.02 ± 0.01ab	nd
Petunidin 3,5-di-*O*-glucoside	A2	nd	nd	nd	nd	nd	nd	0.11 ± 0.01a	0.02 ± 0.00b	nd	nd	0.65 ± 0.03c	0.05 ± 0.01d	0.03 ± 0.00e	nd
Delphinidin 3-*O*-glucoside	A3	nd	nd	nd	nd	nd	nd	nd	nd	nd	nd	0.44 ± 0.02a	nd	nd	nd
Petunidin 3-*O*-glucoside	A4	nd	nd	nd	nd	nd	nd	nd	nd	nd	nd	0.10 ± 0.01a	nd	nd	nd
Other anthocyanins[a]		nd	nd	nd	nd	nd	nd	nd	nd	nd	nd	0.11 ± 0.01a	nd	nd	nd
**Total**		0.04 ± 0.01a	0.02 ± 0.00b	nd	nd	nd	nd	0.38 ± 0.02c	0.10 ± 0.01d	0.06 ± 0.00e	nd	3.72 ± 0.19f	0.13 ± 0.02d	0.05 ± 0.01ae	nd
**Flavonoids**															
Kaempferol sophoroside-glucoside[b]	1	0.09 ± 0.00a	0.05 ± 0.01b	nd	nd	nd	nd	0.22 ± 0.01c	0.35 ± 0.02d	0.13 ± 0.01e	nd	0.20 ± 0.01c	0.26 ± 0.01f	0.16 ± 0.02e	nd
Kaempferol tri-*O*-glucoside[b]	2	2.17 ± 0.11a	2.30 ± 0.12a	0.25 ± 0.01b	nd	nd	nd	6.97 ± 0.35c	7.00 ± 0.36c	3.52 ± 0.18d	0.05 ± 0.00e	6.12 ± 0.31f	6.16 ± 0.37f	5.09 ± 0.25g	1.08 ± 0.05h
Kaempferol tri-*O*-glucoside (acetyl)[b]	3	0.07 ± 0.01a	0.12 ± 0.01b	nd	nd	nd	nd	0.23 ± 0.01c	0.19 ± 0.01d	0.10 ± 0.01b	0.06 ± 0.01a	0.24 ± 0.02c	0.25 ± 0.01c	0.23 ± 0.01c	0.07 ± 0.00a
Quercetin di-*O*-glucoside[c]	5	0.36 ± 0.02a	0.36 ± 0.03a	nd	nd	nd	nd	1.07 ± 0.05b	1.02 ± 0.07b	0.62 ± 0.03c	nd	1.11 ± 0.06b	0.95 ± 0.05b	0.61 ± 0.03c	0.07 ± 0.01d
Quercetin sophoroside[c]	6	1.62 ± 0.08a	1.73 ± 0.09a	0.26 ± 0.02b	nd	nd	nd	4.71 ± 0.24c	4.45 ± 0.22c	2.89 ± 0.14d	0.29 ± 0.01b	4.80 ± 0.24c	4.51 ± 0.27c	3.96 ± 0.20e	1.26 ± 0.06f
Kaempferol 3-*O*-sophoroside	9	19.01 ± 0.95a	19.82 ± 1.19a	4.08 ± 0.20b	nd	nd	nd	54.88 ± 2.74c	54.27 ± 3.25c	36.36 ± 1.82d	4.92 ± 0.25e	61.01 ± 3.05f	55.86 ± 2.79cf	50.17 ± 2.51c	23.54 ± 1.18g
Isorhamnetin 3-*O*-glucoside	16	nd	nd	1.02 ± 0.05a	nd	nd	nd	0.19 ± 0.01b	1.13 ± 0.06a	2.34 ± 0.12c	0.42 ± 0.02d	0.23 ± 0.01e	0.17 ± 0.01b	1.52 ± 0.08f	2.35 ± 0.12c
Quercetin	18	0.10 ± 0.01a	0.23 ± 0.02b	0.11 ± 0.01a	nd	nd	nd	0.73 ± 0.04c	0.59 ± 0.03d	2.46 ± 0.15e	3.13 ± 0.16f	0.72 ± 0.04c	2.10 ± 0.11g	1.53 ± 0.12h	1.53 ± 0.14h
Kaempferol	21	0.78 ± 0.04a	1.53 ± 0.08b	0.87 ± 0.04c	nd	nd	nd	3.07 ± 0.15d	4.47 ± 0.26e	10.08 ± 0.50f	27.69 ± 1.39g	3.52 ± 0.18h	3.47 ± 0.21h	6.91 ± 0.35i	11.15 ± 0.56j
Other flavonoids[b]		3.27 ± 0.16a	4.92 ± 0.25b	1.42 ± 0.07c	nd	nd	nd	10.29 ± 0.51d	10.77 ± 0.64d	10.21 ± 0.61d	8.34 ± 0.42e	11.27 ± 0.56d	10.88 ± 0.54d	10.61 ± 0.74d	6.86 ± 0.41f
**Total**		27.48 ± 1.37a	31.07 ± 1.55b	8.01 ± 0.40c	nd	nd	nd	82.35 ± 4.11d	84.25 ± 5.05d	68.72 ± 3.44e	44.88 ± 2.24f	89.25 ± 4.46d	84.61 ± 5.08d	80.79 ± 4.04d	47.90 ± 2.39f
**TOTAL PHENOLS**		27.52 ± 1.65a	31.08 ± 1.86b	8.01 ± 0.40c	nd	nd	nd	82.73 ± 4.20d	84.35 ± 4.23dg	68.78 ± 3.43e	44.88 ± 2.23f	92.96 ± 4.65g	84.73 ± 4.24dg	80.84 ± 4.85d	47.90 ± 2.40f

§ peak number as reported in Table 2. [a] expressed as delphinidin 3,5-di-*O*-glucoside; [b] expressed as kaempferol 3-*O*-sophoroside equivalents; [c] expressed as quercetin 3-*O*-glucoside equivalents. nd: not detected. Data are given as mean ± standard deviation (n = 3). Mean values within a line with different letters are significantly different (homogenous groups) at $p \leq 0.05$.

(c)

Compounds §		1DES	2DES	3DES	4DES	5DES	6DES	7DES	8DES	9DES	10DES	11DES	12DES	13DES	14DES	15DES	16DES
**Anthocyanins**																	
Delphinidin 3,5-di-*O*-glucoside	A1	4.95 ± 0.25a	10.60 ± 0.53b	8.53 ± 0.43c	11.01 ± 0.66b	10.06 ± 0.50b	10.66 ± 0.53b	12.35 ± 0.62c	11.03 ± 0.55b	10.17 ± 0.51b	8.52 ± 0.43c	4.98 ± 0.25a	8.36 ± 0.42c	7.13 ± 0.36d	2.48 ± 0.12e	6.76 ± 0.34d	3.24 ± 0.16f
Petunidin 3,5-di-*O*-glucoside	A2	1.12 ± 0.06a	1.77 ± 0.09be	1.37 ± 0.07c	1.72 ± 0.09b	1.54 ± 0.08d	1.70 ± 0.10bd	1.94 ± 0.10e	1.71 ± 0.9bd	1.59 ± 0.08bd	1.18 ± 0.06a	0.45 ± 0.03f	1.24 ± 0.06a	1.08 ± 0.06a	0.49 ± 0.02f	1.12 ± 0.06a	0.57 ± 0.04g
Delphinidin 3-*O*-glucoside	A3	0.32 ± 0.02a	0.85 ± 0.04b	0.73 ± 0.04c	1.00 ± 0.06d	0.92 ± 0.04b	1.05 ± 0.05d	1.00 ± 0.07d	0.95 ± 0.05bd	0.84 ± 0.04b	1.05 ± 0.08d	0.83 ± 0.04b	0.91 ± 0.05bd	0.69 ± 0.04c	0.50 ± 0.03e	0.90 ± 0.05bd	0.39 ± 0.02f
Petunidin 3-*O*-glucoside	A4	0.09 ± 0.01a	0.15 ± 0.01bg	0.12 ± 0.01c	0.17 ± 0.01bd	0.15 ± 0.01bg	0.17 ± 0.01bd	0.16 ± 0.01bdg	0.16 ± 0.02bdg	0.14 ± 0.01bc	0.19 ± 0.02d	0.06 ± 0.00e	0.51 ± 0.04f	0.12 ± 0.01c	0.14 ± 0.01cg	0.15 ± 0.01bg	0.11 ± 0.01ac
Other anthocyanins[a]		0.32 ± 0.03a	0.54 ± 0.03b	0.31 ± 0.02a	0.39 ± 0.02c	0.31 ± 0.03a	0.43 ± 0.02c	0.55 ± 0.03b	0.43 ± 0.03c	0.40 ± 0.02c	1.29 ± 0.07d	0.72 ± 0.04e	1.02 ± 0.09f	0.43 ± 0.02c	0.98 ± 0.05f	1.52 ± 0.08g	0.28 ± 0.02a
**Total**		6.80 ± 0.34a	13.91 ± 0.70b	11.07 ± 0.55ch	14.28 ± 0.71b	12.98 ± 0.65be	14.02 ± 0.84b	16.00 ± 0.80d	14.27 ± 0.71b	13.14 ± 0.66be	12.24 ± 0.61e	7.06 ± 0.35a	12.05 ± 0.60ce	9.44 ± 0.56fh	4.59 ± 0.23g	10.46 ± 0.52h	4.58 ± 0.32g
**Flavonoids**																	
Kaempferol sophoroside-glucoside[b]	1	0.23 ± 0.01a	0.30 ± 0.02b	0.17 ± 0.01c	0.15 ± 0.02cd	0.13 ± 0.01d	0.15 ± 0.01cd	0.23 ± 0.02a	0.15 ± 0.01cd	0.14 ± 0.01d	0.07 ± 0.00e	0.25 ± 0.03ab	0.16 ± 0.02cd	0.12 ± 0.02d	0.13 ± 0.01d	0.16 ± 0.02cd	0.26 ± 0.03ab
Kaempferol tri-*O*-glucoside[b]	2	7.07 ± 0.35a	9.10 ± 0.46b	6.04 ± 0.36c	7.55 ± 0.38adf	6.89 ± 0.35d	7.41 ± 0.37adf	8.55 ± 0.43b	7.63 ± 0.40adf	6.98 ± 0.35adf	3.62 ± 0.18e	7.97 ± 0.40f	4.48 ± 0.22g	4.66 ± 0.23g	4.72 ± 0.24g	4.97 ± 0.45g	7.01 ± 0.35a
Kaempferol tri-*O*-glucoside (acetyl)[b]	3	0.23 ± 0.02ad	0.30 ± 0.03b	0.19 ± 0.01cf	0.23 ± 0.01da	0.21 ± 0.02ac	0.25 ± 0.03ad	0.27 ± 0.03abd	0.23 ± 0.02ad	0.21 ± 0.02ac	nd	0.26 ± 0.02bd	nd	0.20 ± 0.02ca	0.11 ± 0.01e	0.17 ± 0.01f	0.14 ± 0.01g
Quercetin di-*O*-glucoside[c]	5	1.21 ± 0.06ac	1.46 ± 0.07b	1.11 ± 0.06a	1.32 ± 0.08bc	1.08 ± 0.05d	1.28 ± 0.06c	1.30 ± 0.07c	1.12 ± 0.07ad	1.04 ± 0.04d	1.47 ± 0.07b	1.11 ± 0.02d	0.30 ± 0.02e	0.09 ± 0.00d	0.09 ± 0.01d	0.93 ± 0.05f	1.27 ± 0.07ac
Quercetin sophoroside[c]	6	5.08 ± 0.25ag	6.60 ± 0.33b	4.53 ± 0.23c	5.41 ± 0.27ad	4.78 ± 0.24c	5.78 ± 0.29de	6.20 ± 0.31be	5.29 ± 0.26a	4.98 ± 0.25ac	2.27 ± 0.12f	4.63 ± 0.22cg	0.87 ± 0.04h	0.92 ± 0.05h	0.77 ± 0.04i	4.04 ± 0.20j	5.70 ± 0.29ae
Kaempferol 3-*O*-sophoroside	9	59.80 ± 2.99aeg	76.95 ± 3.85b	51.91 ± 2.69cg	63.69 ± 3.18ad	58.22 ± 2.91ag	63.56 ± 3.18ad	69.81 ± 3.49d	64.60 ± 3.23de	58.54 ± 2.92aeg	33.53 ± 1.68f	56.96 ± 2.85cg	38.79 ± 1.94h	44.70 ± 2.24i	43.19 ± 2.16i	46.29 ± 2.31i	60.58 ± 3.03ae
Isorhamnetin 3-*O*-glucoside	16	0.16 ± 0.01a	0.24 ± 0.01b	0.21 ± 0.01c	0.20 ± 0.01c	0.17 ± 0.01ae	0.20 ± 0.01c	0.29 ± 0.01d	0.19 ± 0.01ce	0.16 ± 0.02a	4.58 ± 0.23f	3.03 ± 0.15g	2.45 ± 0.12h	0.79 ± 0.04i	1.41 ± 0.07j	0.21 ± 0.01c	0.35 ± 0.02k
Quercetin	18	0.15 ± 0.02a	0.36 ± 0.02b	0.37 ± 0.02b	0.39 ± 0.03bk	0.36 ± 0.02b	0.45 ± 0.02cd	0.49 ± 0.02c	0.46 ± 0.02ck	0.40 ± 0.03bd	3.33 ± 0.17e	0.21 ± 0.01f	1.70 ± 0.09g	0.81 ± 0.05h	1.10 ± 0.06i	0.68 ± 0.04j	0.43 ± 0.03kd
Kaempferol	21	1.60 ± 0.08a	2.02 ± 0.10b	2.09 ± 0.11b	2.03 ± 0.14b	1.84 ± 0.10bc	2.18 ± 0.11b	2.54 ± 0.13d	2.47 ± 0.14d	2.09 ± 0.10b	36.47 ± 1.82e	1.58 ± 0.08a	21.57 ± 1.08f	5.40 ± 0.27g	12.77 ± 0.64h	4.26 ± 0.21i	2.73 ± 0.14d
Other flavonoids[b]		9.89 ± 0.49a	13.62 ± 0.68b	8.54 ± 0.43c	10.82 ± 0.54ade	9.69 ± 0.48a	11.27 ± 0.56de	12.41 ± 0.62d	11.15 ± 0.56e	9.72 ± 0.49a	14.97 ± 0.75bh	18.53 ± 0.93f	21.91 ± 1.31g	15.55 ± 0.78h	20.98 ± 1.05g	25.02 ± 1.25i	11.12 ± 0.78ae
**Total**		85.41 ± 4.27ad	110.95 ± 5.55b	75.15 ± 3.76c	91.78 ± 4.59ad	83.38 ± 4.17a	92.54 ± 4.63ad	102.09 ± 5.10b	93.28 ± 5.59d	84.25 ± 4.89a	100.31 ± 5.02d	94.53 ± 4.73ad	92.22 ± 4.43ad	73.25 ± 3.66c	85.26 ± 4.26a	86.72 ± 5.20a	89.58 ± 4.48a
**TOTAL PHENOLS**		92.21 ± 4.60ad	124.86 ± 6.24b	86.22 ± 4.31af	106.05 ± 5.30ce	96.36 ± 4.82cd	106.56 ± 5.34ce	118.08 ± 5.90b	107.55 ± 5.38e	97.39 ± 5.87cd	112.55 ± 5.63be	101.59 ± 6.09cde	104.27 ± 5.21ce	82.69 ± 1.13f	89.86 ± 5.39ad	97.18 ± 4.86d	94.16 ± 5.18ad

§ peak number as reported in Table 2. [a] expressed as delphinidin 3,5-di-*O*-glucoside; [b] expressed as kaempferol 3-*O*-sophoroside equivalents; [c] expressed as quercetin 3-*O*-glucoside equivalents. nd: not detected. Data are given as mean ± standard deviation (n = 3). Mean values within a line with different letters are significantly different (homogenous groups) at $p \leq 0.05$.

Table S2. Regression coefficient of polynomial function of the most significant response surfaces during UAE

Term [a]	Coefficients	Standard Error	*F*-Value	*p*-Value [b]
delphinidin 3,5-di-O-glucoside				
Intercept	74.52	1.10		
X_1	-2.01	0.8709	5.34	0.0541
X_2	2.51	0.8709	8.32	0.0235
X_3	1.25	0.8709	2.06	0.1943
X_1X_2	-1.72	1.23	1.96	0.2041
X_1X_3	-1.55	1.23	1.58	0.2485
X_2X_3	-0.7000	1.23	0.3230	0.5875
X_1^2	-9.62	1.20	64.26	< 0.0001
X_2^2	-1.47	1.20	1.50	0.2596
X_3^2	0.9025	1.20	0.5653	0.4767
kaempferol 3-O-sophoroside				
Intercept	7.34	0.4642		
X_1	2.60	0.3670	50.19	0.0002
X_2	0.1250	0.3670	0.1160	0.7434
X_3	0.1250	0.3670	0.1160	0.7434
X_1X_2	0.1500	0.5190	0.0835	0.7809
X_1X_3	0.3500	0.5190	0.4548	0.5217
X_2X_3	0.1000	0.5190	0.0371	0.8527
X_1^2	-4.97	0.5059	96.53	< 0.0001
X_2^2	0.2800	0.5059	0.3064	0.5971
X_3^2	0.3300	0.5059	0.4256	0.5350

Table S3. Analysis of variance (ANOVA) of the selected modelled responses during UAE

Source	Sum of Squares	Degree of Freedom	Mean Square	*F*-Value	*p*-Value
delphinidin 3,5-di-O-glucoside					
The recovery					
Model	525.62	9	58.40	9.63	0.0035
Residual	42.47	7	6.07		
Lack of fit	33.50	3	11.17	4.98	0.0775
Pure error	8.97	4	2.24		
Total	568.09	16			
$R^2 = 0.9252$					
kaempferol 3-O-sophoroside					
The recovery					
Model	158.98	9	17.66	16.39	0.0006
Residual	7.54	7	1.08		
Lack of fit	0.0700	3	0.0233	0.0125	0.9978
Pure error	7.47	4	1.87		
Total	166.52	16			
$R^2 = 0.9547$					

-

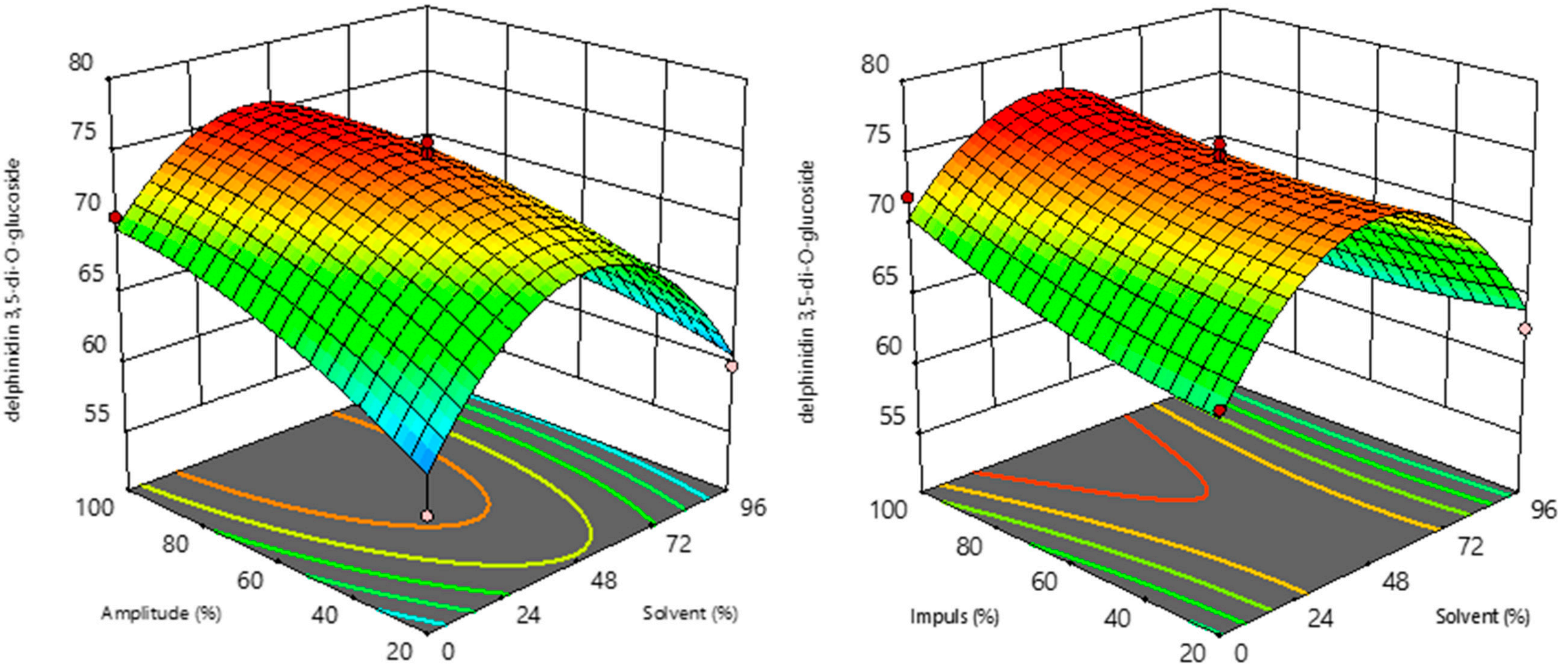

Figure S1. Three-dimensional plots for obtained delphinidin 3,5-di-*O*-glucoside in extracts as a function of UAE process parameters.

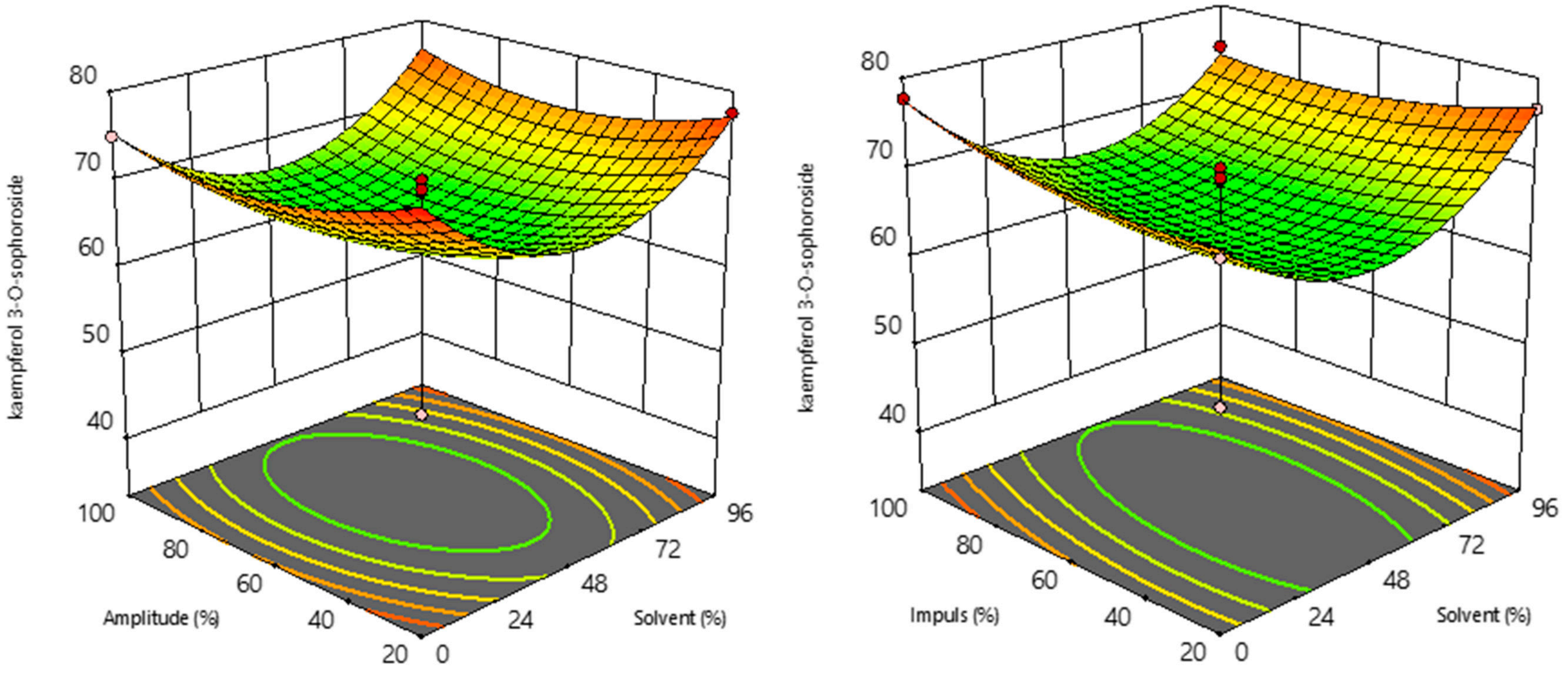

Figure S2. Three-dimensional plots for obtained kaempferol 3-*O*-sophoroside in extracts as a function of UAE process parameters.
